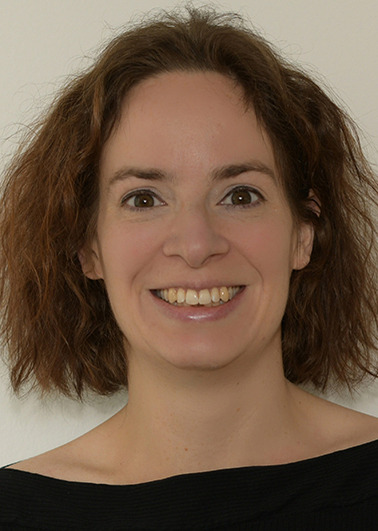# Chiral Phosphoric Acids as Versatile Tools for Organocatalytic Asymmetric Transfer Hydrogenations

**DOI:** 10.1002/ejoc.202100894

**Published:** 2021-10-14

**Authors:** Ádám Márk Pálvölgyi, Fabian Scharinger, Michael Schnürch, Katharina Bica‐Schröder

**Affiliations:** ^1^ Institute of Applied Synthetic Chemistry TU Wien Getreidemarkt Vienna, 9/163 1060 Wien Austria

**Keywords:** Asymmetric Transfer Hydrogenation, Catalysis, Chiral Phosphoric Acids, Metal-Free Hydrogenation, Organocatalysis

## Abstract

Herein, recent developments in the field of organocatalytic asymmetric transfer hydrogenation (ATH) of C=N, C=O and C=C double bonds using chiral phosphoric acid catalysis are reviewed. This still rapidly growing area of asymmetric catalysis relies on metal‐free catalysts in combination with biomimetic hydrogen sources. Chiral phosphoric acids have proven to be extremely versatile tools in this area, providing highly active and enantioselective alternatives for the asymmetric reduction of α,β‐unsaturated carbonyl compounds, imines and various heterocycles. Eventually, such transformations are more and more often used in multicomponent/cascade reactions, which undoubtedly shows their great synthetic potential and the bright future of organocatalytic asymmetric transfer hydrogenations.

## Graphical abstract

Organocatalytic asymmetric transfer hydrogenation provides a metal‐free and ambient alternative to classical reductions. Chiral phosphoric acid‐catalysis represents a powerful strategy in this field, with a range of novel and selective methods for various double bond reductions. This Minireview provides an overview on recent advances including the effect of different phosphoric acids and reductants.

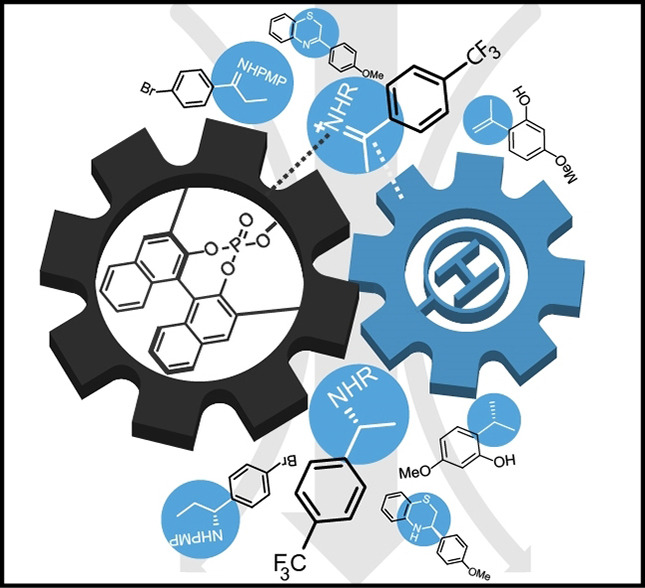

## 1 Introduction

The synthesis of optically active products by asymmetric catalysis plays a crucial role in modern chemistry.^[1]^ In this field, the asymmetric hydrogenation of unsaturated compounds is of special interest, featuring an extreme broad range of industrial applications.^[2]^ Inspired by natural oxidoreductases, the introduction of 1,4‐dihydropyridines (Hantzsch esters, **HE**s) as biomimetic hydrogen sources opened the era of organocatalytic asymmetric transfer hydrogenations (ATH).[^3^, ^4^] As neither gaseous H_2_, nor expensive and/or toxic metal sources are required, such asymmetric transformations show great air and moisture tolerance which results in operational simplicity and additionally safer processes. In light of the growing awareness for metal‐free, safe and sustainable chemical reactions, the field of organocatalytic ATH received rapidly increasing attention in the last 15 years.

Since the pioneering discoveries by the group of Terada^[5]^ and Akiyama,^[6]^ chiral phosphoric acids (**CPA**s) are amongst the most powerful organocatalysts, and they found a tremendously broad range of applications in organo‐ and transition‐metal‐catalysis.[^7^, ^8^, ^9^]

**Figure 1:**
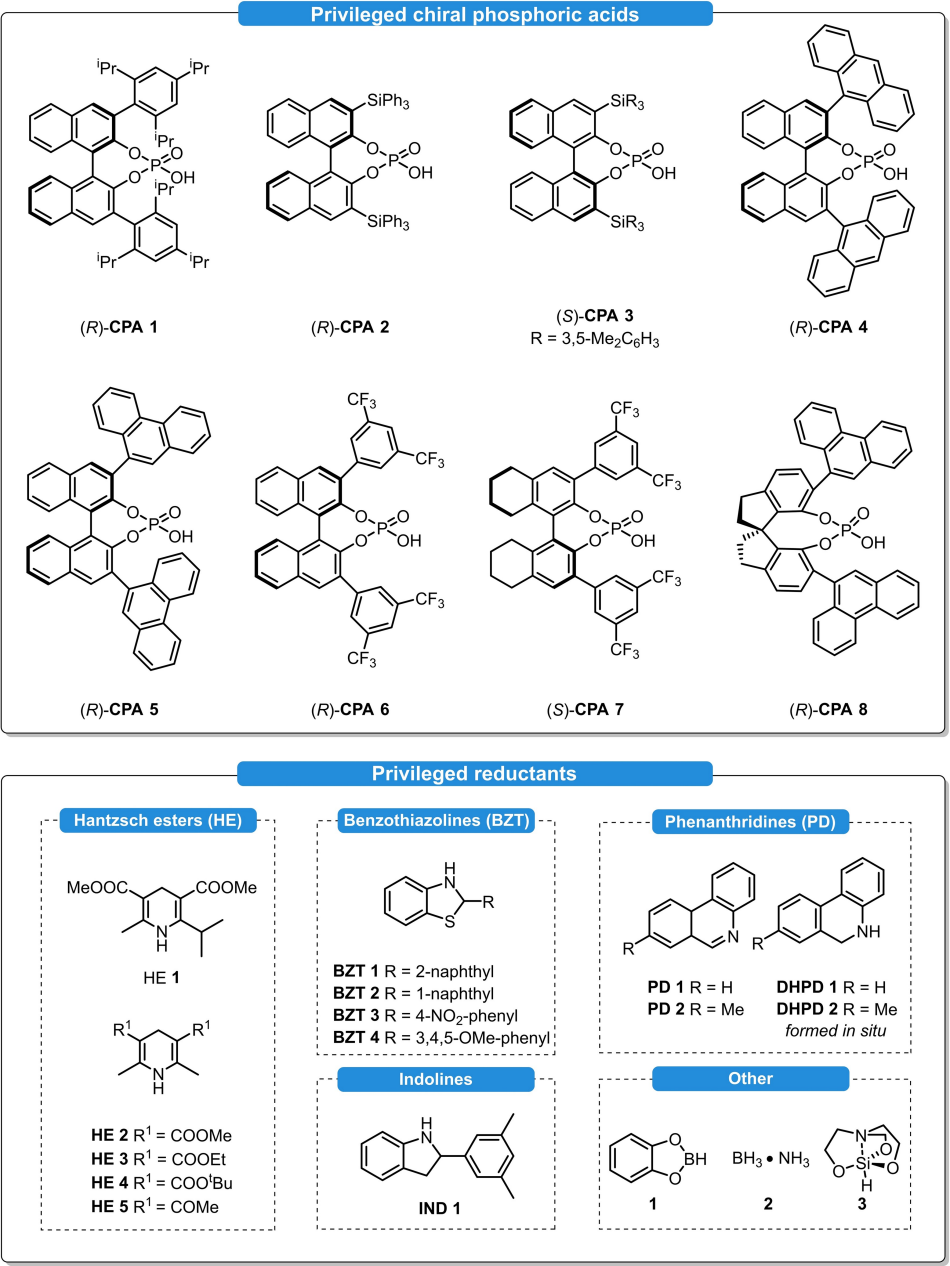
The most typically used chiral phosphoric acids (top) and hydrogen sources (bottom) for asymmetric transfer hydrogenations.

While Hantzsch dihydropyridines (**HE**s) are indeed to date the most frequently used hydrogen sources for organocatalytic ATH reactions,[^10^, ^11^, ^12^] a few alternative reductants have been developed as well. Bearing a substituent geminal to the transferable hydrogen, benzothiazolines (**BZT**s) and indolines (**IND**s) are highly tunable hydrogen sources as their catalytic activity and selectivity highly depends on the fine tuning of their electronic and steric properties.[^13^, ^14^] Moreover; ATH reactions using other NADH‐analogues like phenanthridines (**PD**s) as well as borane‐mediated ATH reactions in combination with CPAs have been also reported. An overview of the most frequently used chiral phosphoric acids and hydrogen sources is depicted in Figure 1.

**Scheme 1:**
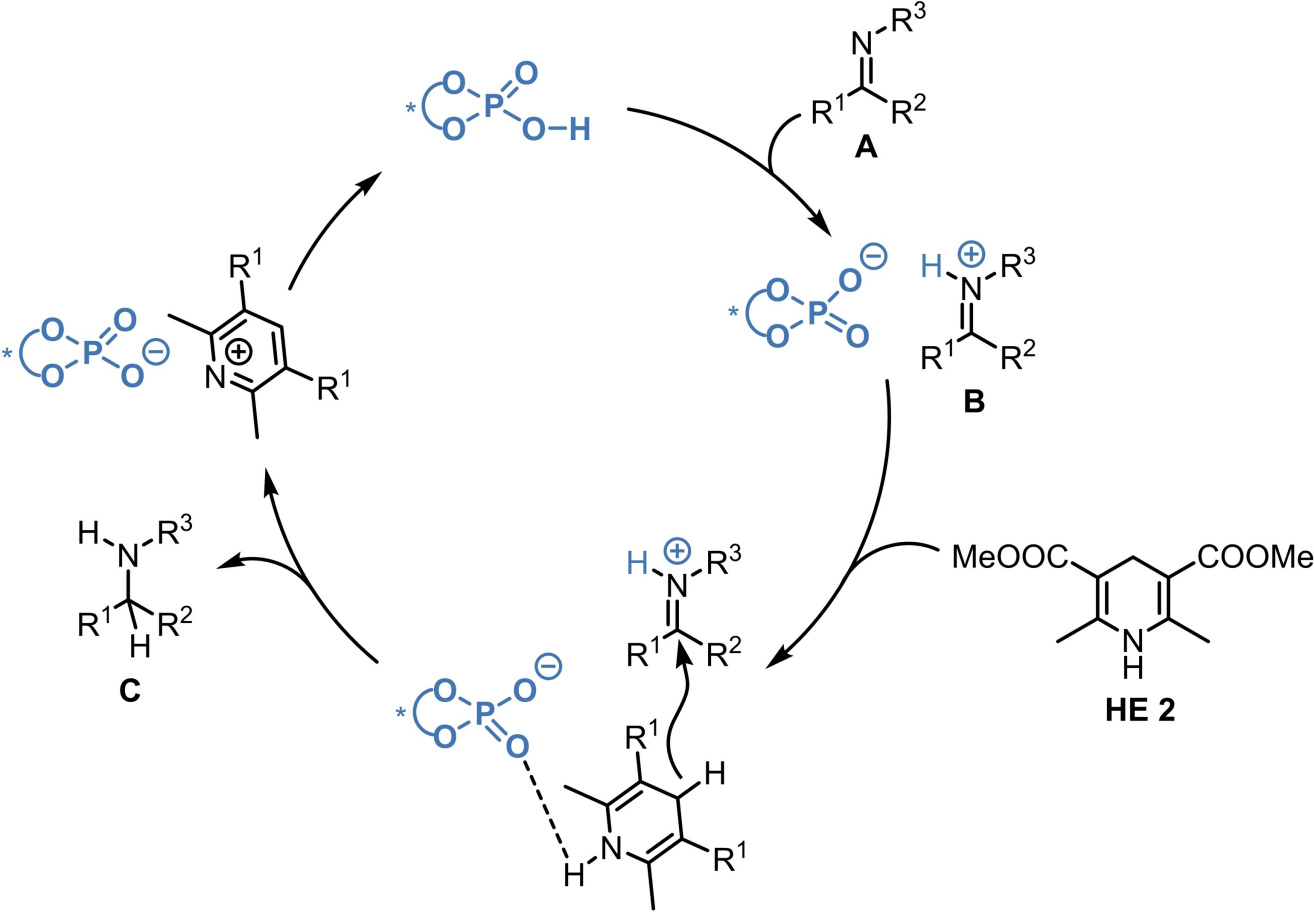
Typical reaction mechanism of CPA‐catalyzed asymmetric transfer hydrogenations, illustrated with the ATH of ketimine **A**.

The typical reaction mechanism of such asymmetric transfer hydrogenations is illustrated with the ATH of ketimine **A** and **HE 2** (Scheme 1). The phosphoric acid acts both as a Brønsted‐acid and as a Lewis‐base therefore it plays a dual role in the reaction: it activates the substrates *via* protonation generating the iminium species **B**; meanwhile, as a H‐bond acceptor it can also direct the hydrogen source, facilitating the subsequent hydride transfer. After the formation of the iminium species **B**, the hydride transfer yields the enantioenriched amine product **C** and the pyridinium salt, which can undergo proton transfer to regenerate the phosphoric acid (Scheme 1).

This Minireview aims to give an overview on the field of chiral phosphoric acid catalyzed asymmetric organocatalytic ATH reactions with emphasis on recent developments since 2014.

## 2 Reduction of C=C and C=O Double Bonds

**Scheme 2:**
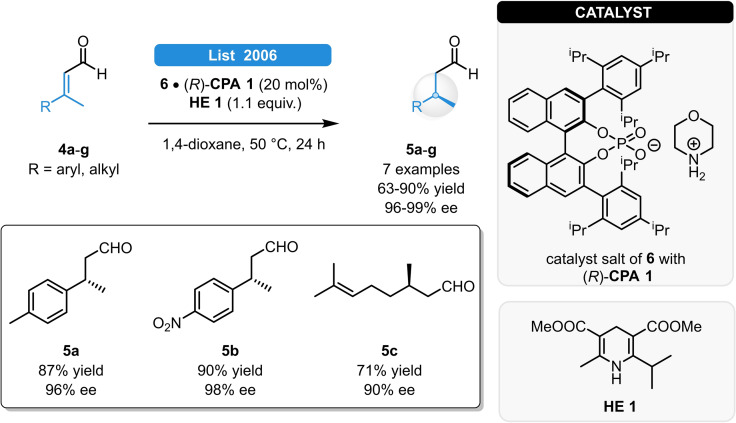
ATH of enals *via* asymmetric counteranion directed catalysis.

Independently and parallel with the developments of iminium catalysis, in 2005, Mayer and List reported an alternative approach for the ATH of enals (Scheme 2).^[15]^ The combination of morpholine (**6**) with (*R*)‐**CPA 1** (also known as (*R*)‐TRIP) resulted in high catalytic activities and enantioselectivity for the ATH of 3‐alkylcinnamaldehyde derivatives (**5 a**–**g**). Moreover, sterically non‐hindered aliphatic substrates could be also efficiently reduced. The reaction was found to be stereoconvergent as the pure (*E*)‐ and (*Z*)‐isomer, as well as the diastereomeric mixture of the enal substrates all resulted in the same product antipode.

**Scheme 3:**
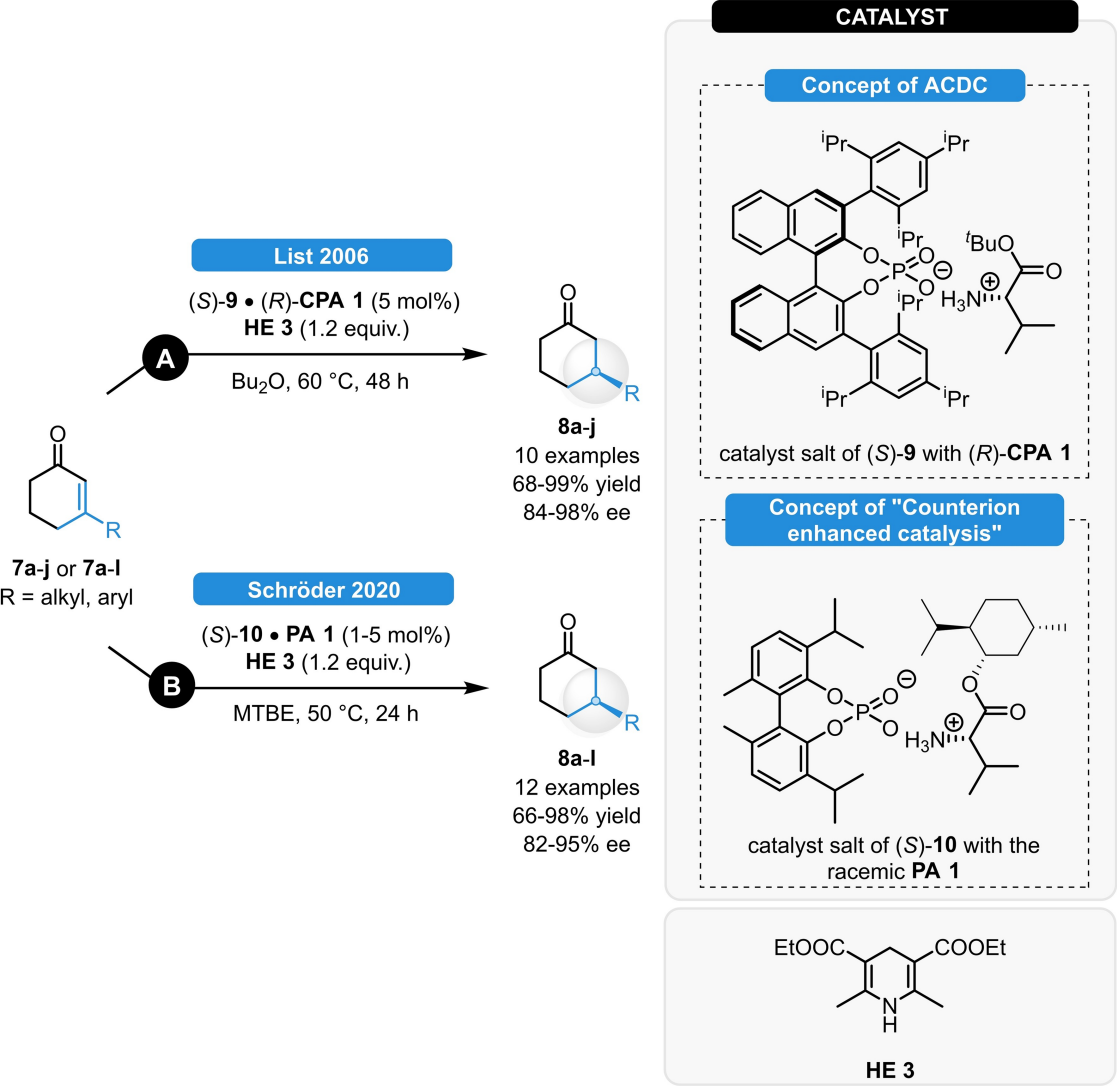
ATH of cyclic enones *via* asymmetric counteranion directed catalysis (**A**) and *via* “counterion enhanced catalysis” (**B**).

The same catalytic concept could be later extended to the ATH of enones (Scheme 3, top).^[16]^ The catalyst salt of l‐valine *tert*‐butyl ester ((*S*)‐**9**), together with the chiral phosphoric acid (*R*)‐**CPA 1** provided >90 % yields and excellent enantioselectivity for the ATH of various 3‐substituted cyclic enones (**7 a**–**j**). The (*S*)‐**CPA 1** gave significantly inferior results, indicating a sharp difference between the matched and mismatched ion‐pairing. Based on these discoveries, the general concept of asymmetric counteranion directed catalysis (ACDC) emerged; referring to any asymmetric catalytic reaction in which the enantiodiscrimination is induced through the tight ion‐pairing of a cationic intermediate with an enantiomerically pure anion.^[17]^

We recently showed that the novel concept of “Counterion Enhanced Catalysis” can be also very effective for asymmetric transfer hydrogenations (Scheme 3, bottom).^[18]^ As a conceptual spin‐off of the ACDC approach, we relied on rather simple and cheap chiral frameworks composing of amino‐acid esters, in combination with achiral or racemic phosphoric acids. After optimization, using the catalyst salt of (*S*)‐**10** with the isopropyl‐substituted, racemic **PA 1**, similarly high yields and enantioselectivities (82–95 % ee) as compared to ACDC for the ATH of various cyclic enones were observed. Given the significantly cheaper, easier and faster catalyst synthesis, as well as the natural origin of both catalyst components; this methodology could provide a valuable alternative to the current state‐of‐the‐art and it might be also a useful tool for a significantly broader range of future applications.

**Scheme 4:**
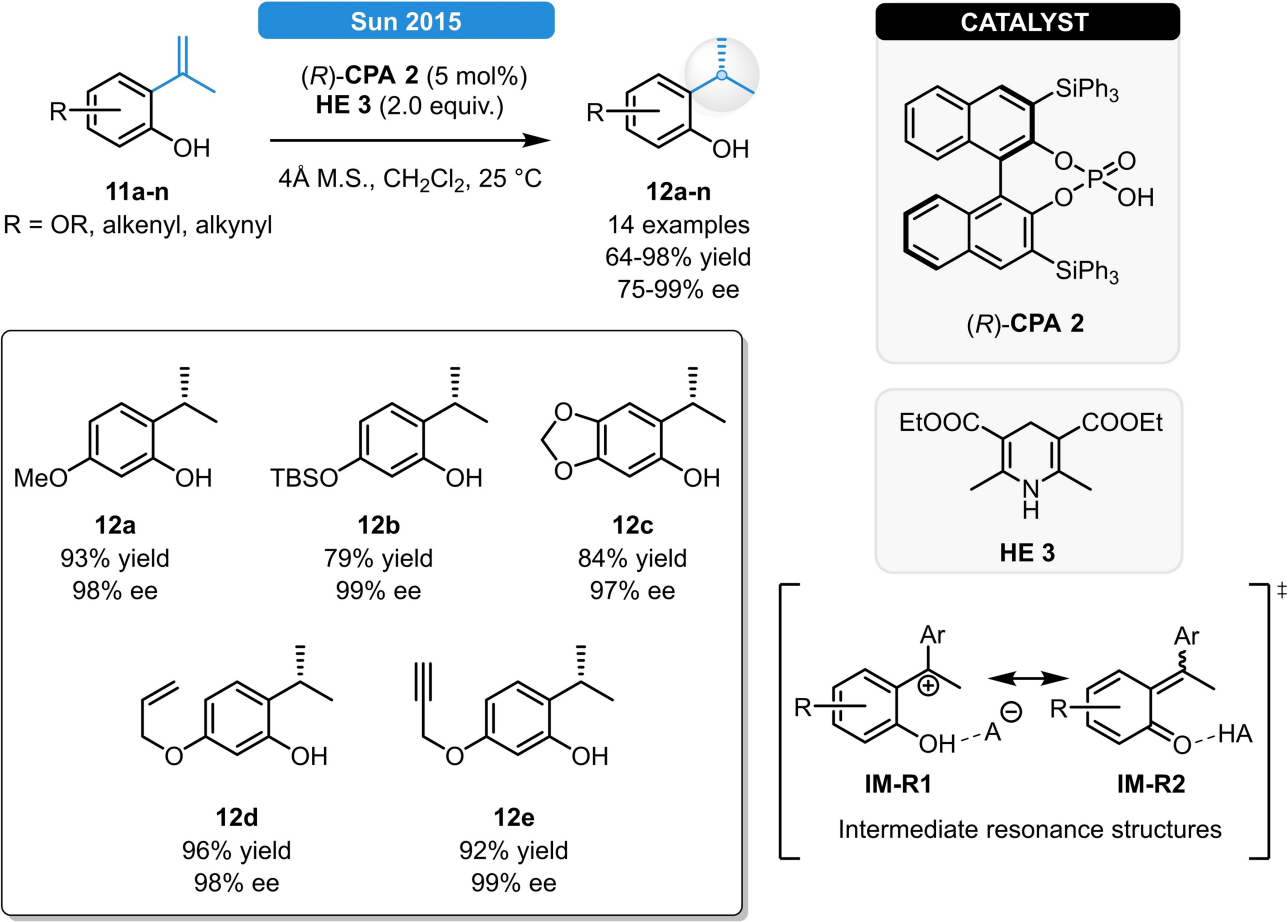
ATH of electron rich stryrenes (left) and the plausible intermediates (right bottom).

In 2015 Sun *et al*. reported an elegant strategy for the ATH of electron rich terminal double bonds (Scheme 4). The phosphoric acid (*R*)‐**CPA 2**, together with Hantzsch ester **HE 3** provided a convenient access to 1,1‐diarylethanes (**12 a**–**n**) in excellent yield and up to >99 % ee.^[19]^ Full chemoselectivity towards the reduction of the terminal alkene bond was observed as various functional groups including silyl ethers, alkenes, alkynes and thioethers were all well tolerated (Scheme 4). As significantly worse results were obtained for styrene substrates without the *ortho*‐hydroxyl functionality, the phenolic OH‐group is believed to act as a directing group: initial protonation of the substrate **11 a**–**n** results in the formation of an intermediate – described with its extreme resonance **IM‐R1** and **IM‐R2** forms – in which the (*R*)‐**CPA 2** remains in close proximity to the substrate by means of H‐bonding with the OH‐group as a key factor for the high stereocontrol. DFT calculations showed that even though the (*Z)*‐isomer is less stable, it forms preferentially, which was in accordance with the experimentally observed absolute stereochemistry.

**Scheme 5:**
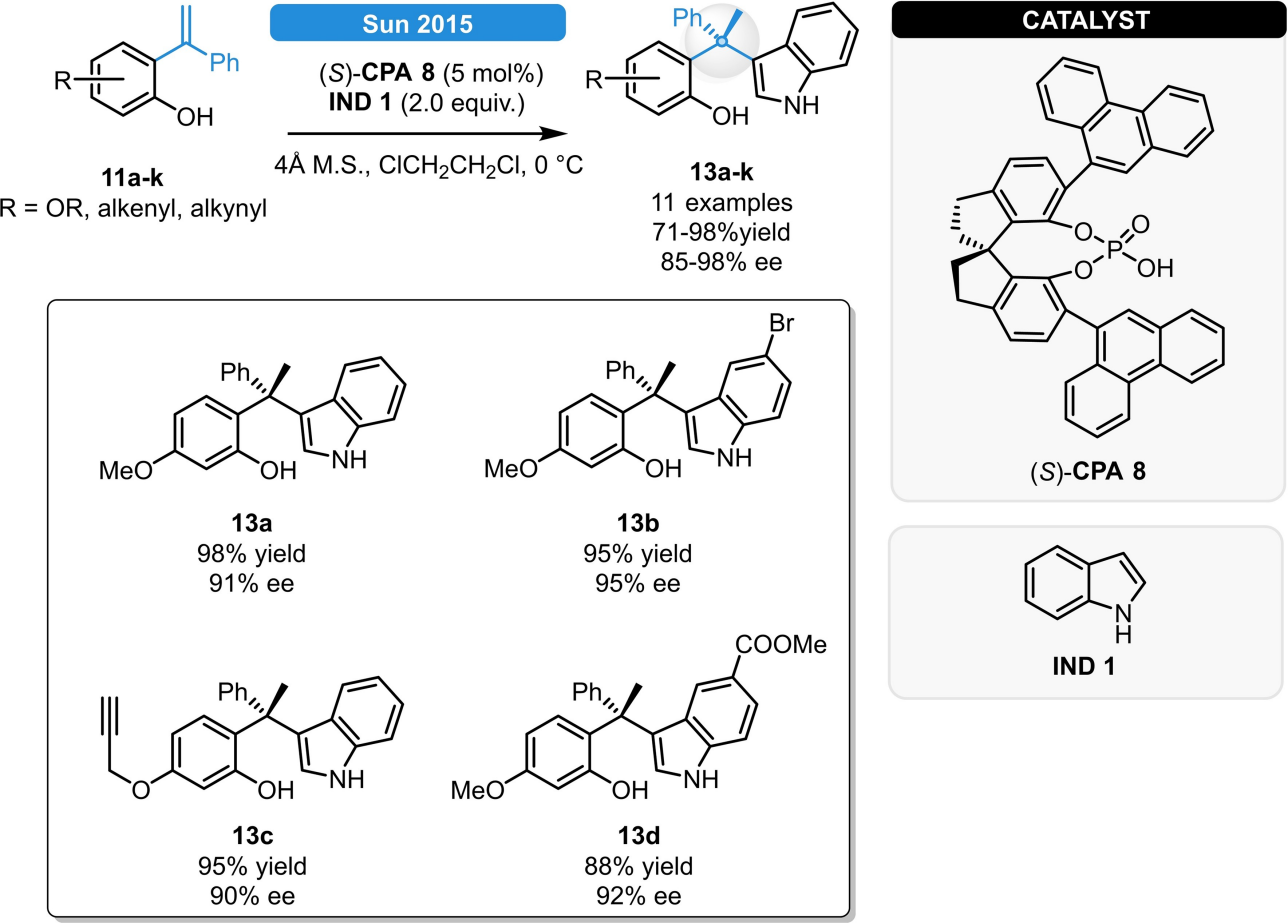
Asymmetric hydroarylation of electron rich stryrenes.

When replacing Hantzsch esters with indole nucleophiles, asymmetric hydroarylations could be carried out with high level of stereocontrol (Scheme 5).^[19]^ The best results were obtained with the SPINOL‐derived phosphoric acid (*S*)‐**CPA 8** resulting in **13 a**–**k** in >90 % yields and ee. Eventually, the potential biological activity for either of the product classes was evaluated based on their cytotoxicity on human lung cancer cells, providing IC_50_ values in the low micromolar region.

**Scheme 6:**
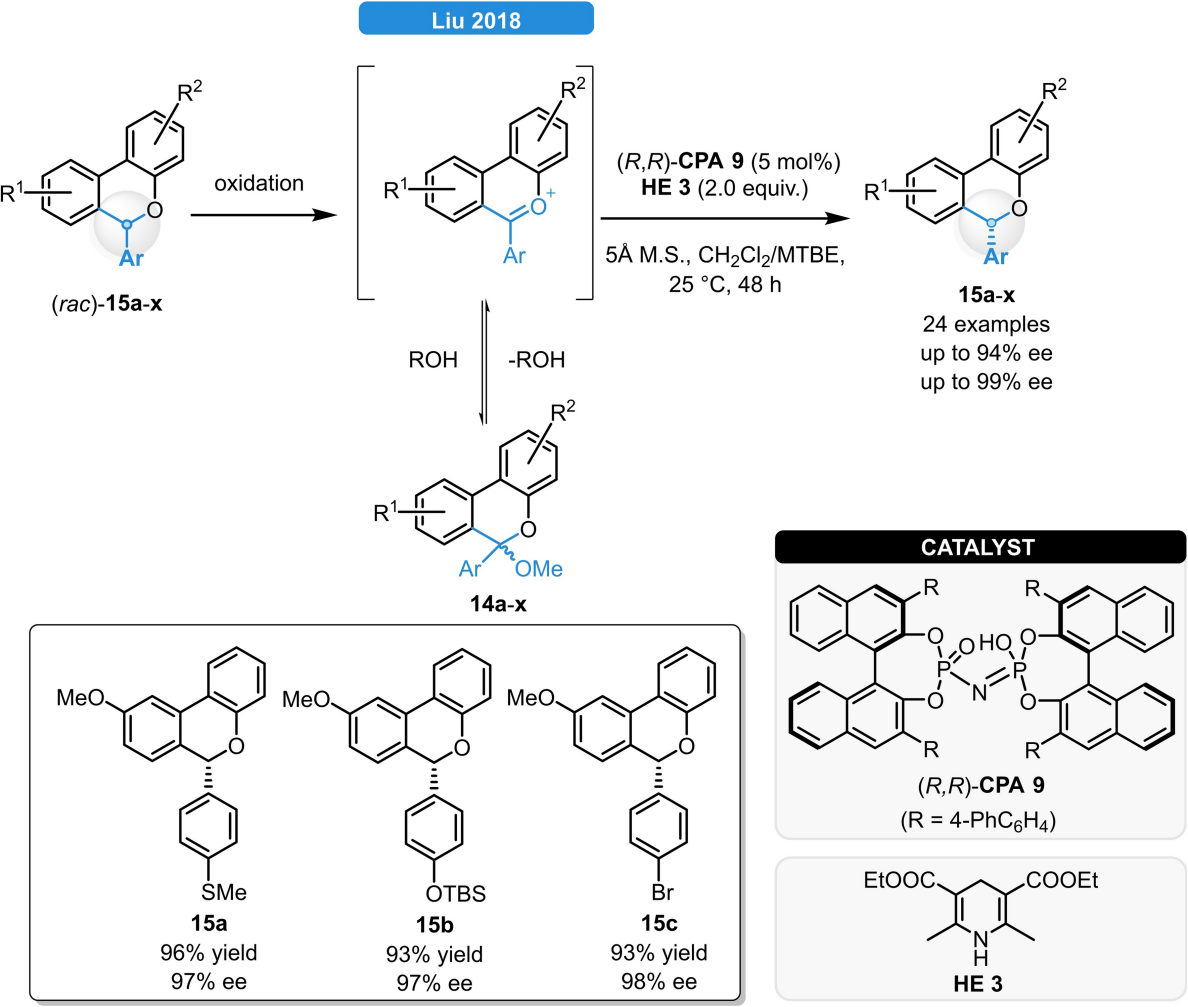
*C*_2_‐symmetric imidodiphosphoric acid‐catalyzed ATH of aromatic ketals.

The group of Liu reported a highly enantioselective method for the synthesis of 6*H*‐benzo[c]chromenes *via* the ATH of ketals by a redox‐deracemization strategy (Scheme 6):^[20]^ The oxidation of racemic **15 a**–**x** and the subsequent enantioselective oxocarbenium‐reduction led to the formation of various chromenes. While traditional CPA‐catalysis failed to achieve high enantioselectivity and resulted only in 10–55 % ee, the *C*_2_‐symmetric imidodiphosphoric acid (*R*,*R*)‐**CPA 9** in the solvent mixture of CH_2_Cl_2_ and MTBE provided excellent stereoselectivity for the ATH of *rac*‐**15 a**. Under the optimized reaction conditions, various α‐substituted 6*H*‐benzo[c]chromenes bearing different substituents on either of the aromatic rings (*rac*‐**15 a**–**x**) could be readily reduced in 86–97 % ee.

**Scheme 7:**
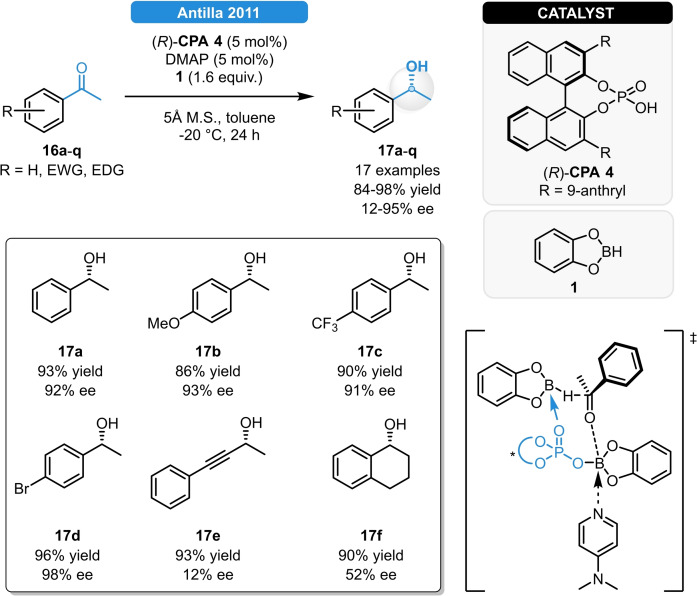
CPA‐catalyzed asymmetric transfer hydrogenation of prochiral ketones.

The asymmetric reduction of C=O double bonds is predominantly achieved *via* transition‐metal‐catalysis;[^21^, ^22^, ^23^, ^24^, ^25^] however, a few organocatalytic variants were also reported in the last decade. Antilla and co‐workers developed the first CPA‐catalyzed ATH reaction of prochiral ketones.^[26]^ The phosphoric acid (*R*)‐**CPA 4**, in combination with catecholborane and DMAP resulted in excellent enantioselectivity for the reduction of acetophenone derivatives bearing electron donating and withdrawing groups; meanwhile, moderate selectivity was observed for aliphatic analogues (e. g.: **17 e**). Based on ^11^B NMR studies, it is believed that a chiral boron species is formed. As this features both a Lewis acidic and a Lewis basic moiety, it can simultaneously coordinate to the DMAP and increase the nucleophilicity of an unreacted catecholborane, resulting in the transition state depicted in Scheme 7. Very recently, the authors reported a similar strategy for the C=C reduction of *trans*‐chalcones, resulting in 78–96 % ee.^[27]^

**Scheme 8:**
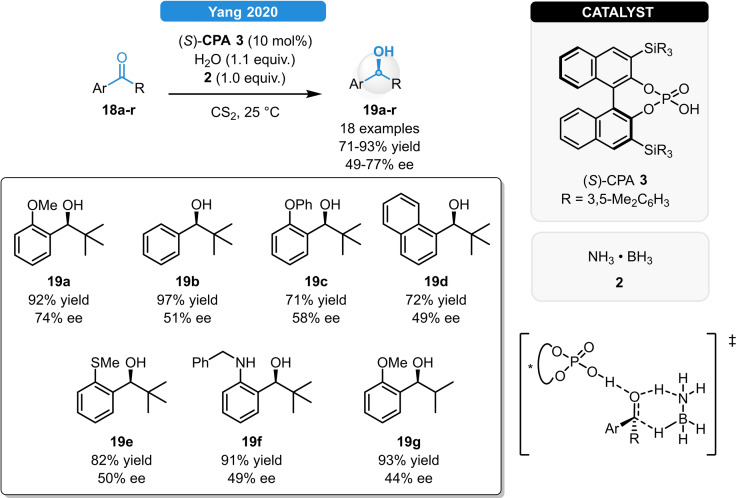
A CPA‐catalyzed asymmetric transfer hydrogenation of bulky aryl ketones.

Recently, Yang *et al*. successfully applied CPAs for the asymmetric reduction of bulky aryl ketones using ammonia borane as hydrogen source (Scheme 8). The corresponding secondary alcohols (**19 a**–**r**) were obtained in high yields and in moderate enantioselectivities of 43–77 % ee. The CPA could be *in situ* continuously regenerated with the assistance of water and excess of ammonia borane allowing to use only 0.5 mol% of (*S*)‐**CPA 3**. According to DFT studies, the phosphoric acid promotes the double hydrogen transfer between the ammonia borane and the ketone, as the transfer of the hydridic and protic hydrogens take place simultaneously through a pericyclic six‐membered transition state as a source of asymmetric induction.^[28]^

## 3 Reduction of C=N Double Bonds

Since the independent pioneering works of Rueping,^[29]^ List,^[30]^ and MacMillan;^[31]^ the synthesis of optically active amines and *N*‐heterocycles with the aid of CPAs represents the most extensively studied field of organocatalytic ATH reactions. Herein, recent advancements starting from 2014 will be discussed.

### 3.1 The ATH of imine derivatives

**Scheme 9:**
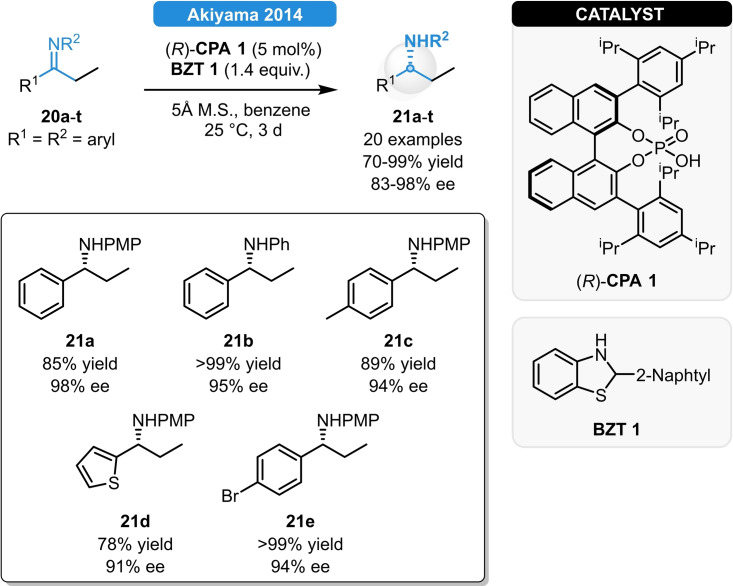
Asymmetric transfer hydrogenation of ethyl ketimines.

Based on their previous reports with benzothiazoline‐type hydrogen donors, the group of Akiyama reported the ATH of ethyl ketimines as an extension of their previous studies.^[32]^ While traditional Hantzsch esters failed to achieve high yields and enantioselectivity as only up to 53 % ee was observed, benzothiazolines provided much better results. After fine tuning the reductant's steric properties, a series of ethyl ketimines (**20 a**–**t**) could be reduced in 70–99 % yield and in 90–98 % ee (Scheme 9). The different catalytic efficiency of the reductants was examined *via* DFT calculations. This revealed that unlike Hantzsch esters, benzothiazolines exert a significant substituent effect which results in an increased energy difference between the diastereomeric transition states, resulting in significantly higher ee. Eventually, the reaction scope was extended to the reductive amination of aliphatic ethyl ketones, resulting in 70–86 % yields and 72–97 % ee. Using the same concept, indolines bearing sterically demanding 2‐substituents were also found to be suitable hydrogen donors and their catalytic efficiency was demonstrated in the ATH of ketimines as well as for the reductive amination of aliphatic ketones, providing excellent stereocontrol for both substrate classes; meanwhile, the sacrificial hydrogen source could be regenerated *via* treatment with tin powder and HCl in EtOH in 92 % yield.^[33]^

**Scheme 10:**
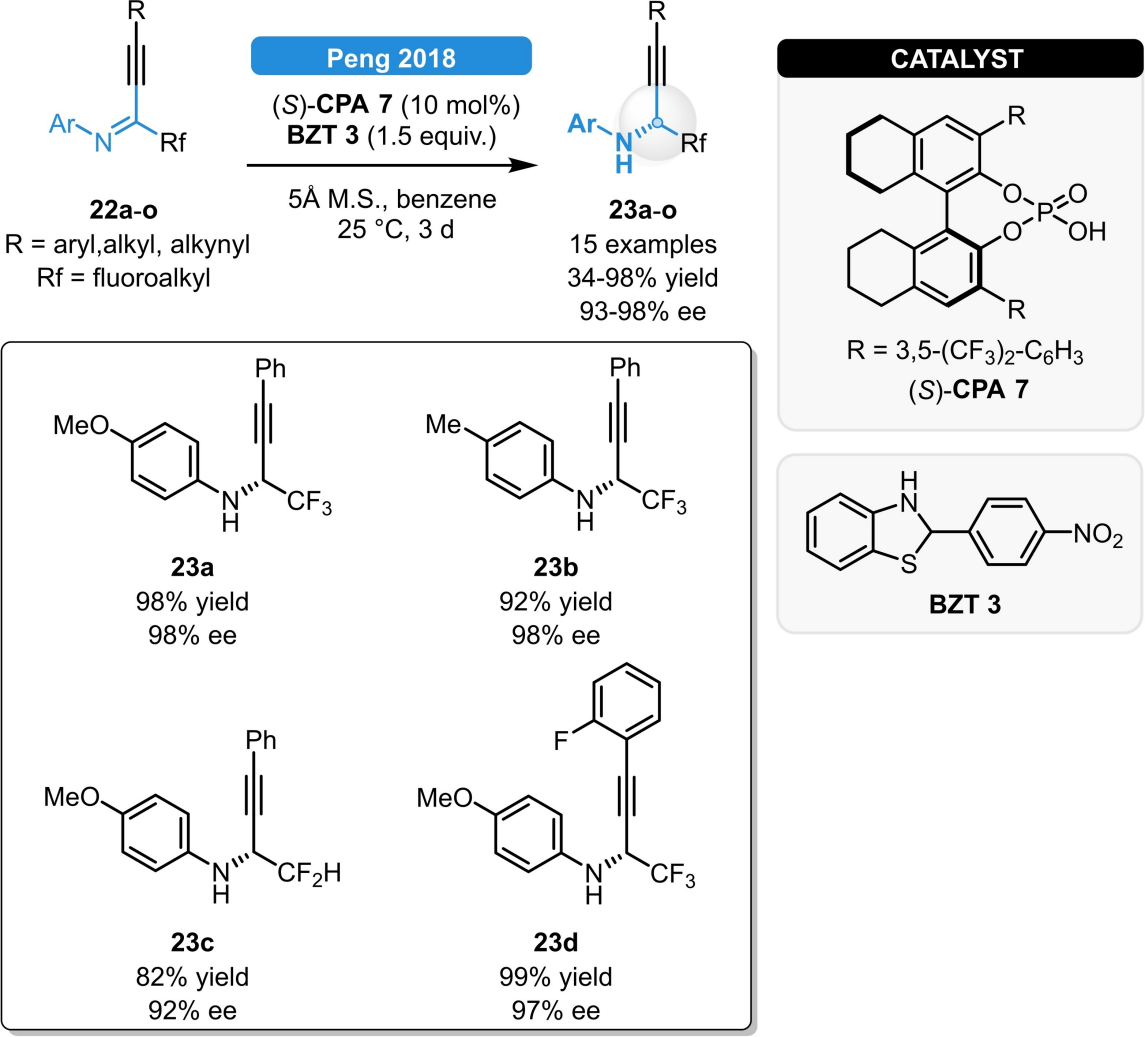
Asymmetric transfer hydrogenation of fluorinated alkynyl ketimines.

Benzothiazolines were also found to be suitable hydrogen sources for the ATH of alkynyl imines. Peng *et al*. reported a CPA‐catalyzed strategy for the synthesis of fluorinated propargylamines (Scheme 10). The best results were obtained using the combination of (*S*)‐**CPA 7** and **BZT 3** bearing a *para‐*nitrophenyl substituent, as substrates with different electronic properties (**22 a**–**o**) could be reduced with 82–98 % yield and >90 % ee. Moreover, the propargylamine **23 a** was found to be a valuable intermediate for the synthesis of dihydroquinolines.^[34]^ A similar approach was reported by the group of Akiyama for the ATH of aryl‐alkynyl‐substituted imines, as a small pool of α‐trifluoromethyl propargylamines was prepared in excellent, 93–98 % enantioselectivity.^[35]^ Both procedures were chemoselective as not even partial reduction of the C≡C bond was observed; moreover, the nature of the hydrogen donor was found to be crucial as no product formation was observed with Hantzsch esters in neither cases.

**Scheme 11:**
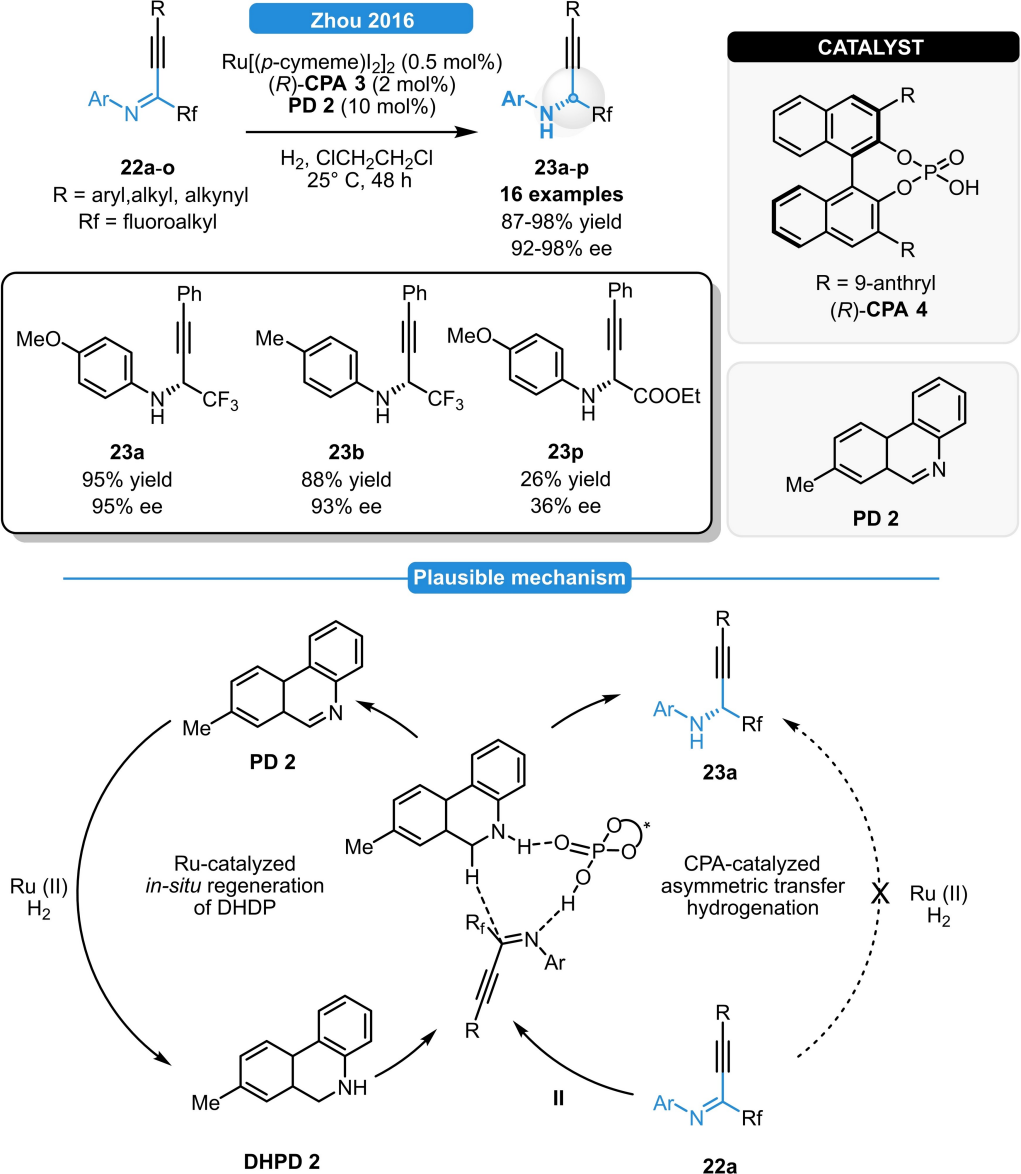
Asymmetric transfer hydrogenation of fluorinated alkynyl ketimines *via* Ru/CPA combined catalysis.

The chemoselective ATH of aryl‐alkynyl‐imines could be also performed *via* biomimetic reduction relying on Ru/chiral phosphoric acid catalysis in the presence of H_2_ as terminal hydrogen source (Scheme 11).^[36]^ In 2016, Zhou *et al*. reported a highly efficient procedure for the synthesis of fluorinated propalgylamines by such elegant method, which also allowed to use only a catalytic amount of biomimetic reductant. After parameter optimization, various aromatic propalgylimines (e. g.: **22 a**–**b**) could be successfully reduced in high yields and in 92–98 % ee by using catalytic amount of Ru[(*p*‐cymeme)I_2_]_2_, (*R*)‐CPA **4** and phenanthridine PD **2** in the presence H_2_ as terminal hydrogen source. The reaction was found to be completely chemoselective to C=N reduction; meanwhile, the importance of the Rf‐group became also evident as the **23 p** was obtained only in moderate yield and ee. The plausible reaction mechanism comprises of two cycles. At first, **PD 2** undergoes reduction *via* Ru/H_2_, affording **DHDP 2**. Then, the ATH of the substrate (e. g.: **22 a**) with **DHDP 2** in the presence of the organocatalyst (*R*)‐**CPA 4** provides the propalgylamine products (e. g.: **23 a**). The high level of asymmetric induction could be attributed to the extreme slow direct substrate reduction *via* Ru/H_2_.

**Scheme 12:**
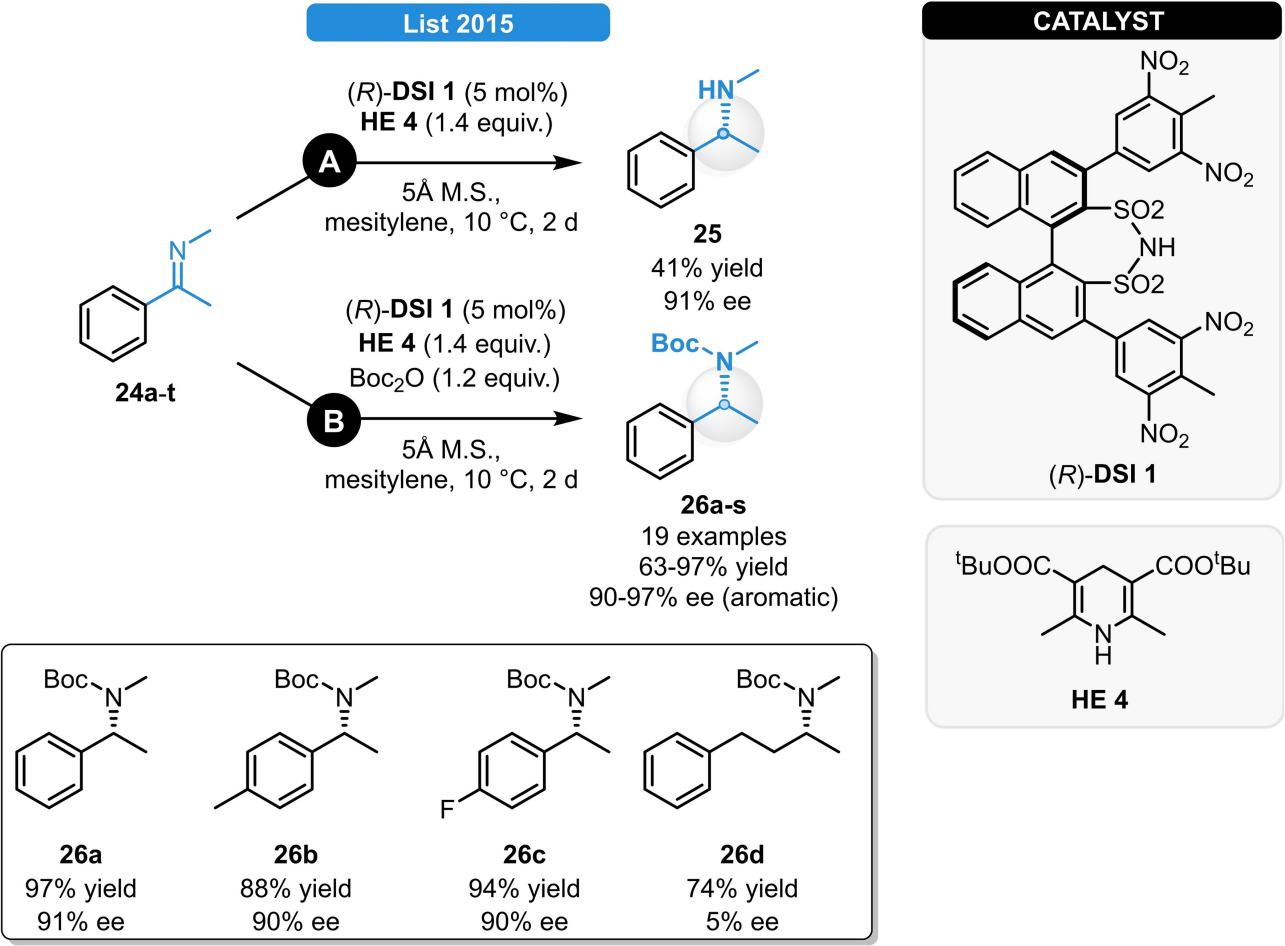
Chiral DSI‐catalyzed asymmetric transfer hydrogenation of *N*‐alkyl imines.

While the aforementioned strategies all required an *N*‐aryl imine for high levels of asymmetric induction, several advances have been reported later for the ATH of *N*‐alkyl analogues (Scheme 12). Addressing this issue, List and co‐workers initially investigated the ATH of *N*‐methyl imine **24 a**.^[37]^ Only moderate catalytic activities and poor enantioselectivity (<30 % ee) was observed when using CPA catalysis; however, this was significantly improved when a chiral disulfonimide (DSI) was applied instead. The Brønsted acid (*R*)‐**DSI 1** afforded the desired product in excellent stereocontrol, albeit only in a moderate yield of 41 % as the salt formation with the product **25** resulted in continuous catalyst deactivation. (Scheme 12, pathway **A**). In order to solve this problem, the amine product **25** was *in situ* derivatized with Boc_2_O (Scheme 12, pathway **B**), yielding **26 a** in excellent yield and ee. With this latter method, various aromatic *N*‐alkyl imines could be successfully reduced in high yields and enantioselectivities (**26 a**–**s**); however, *ortho* and *meta* substitutions and aliphatic groups were not well tolerated, resulting in close‐to‐racemic products (e. g.: **26 d**). Providing synthetic utility for the optically active products, APIs like the antianginal (*R*)‐Fendiline as well as (*S*)‐Rivastigmine, a drug for Parkinson treatment were also synthesized. Based on the same methodology, the reduction of *N*‐alkyl aryl imino esters was realized by Marsden *et al*. After the subsequent removal of the Boc‐group, various *N*‐alkylated arylglycines were synthetized in 67–95 % yield and 50–90 % ee. Offering a great synthetic potential, diverse peptide building blocks and *N*‐heterocycles were also prepared after further derivatizations.^[38]^ Detailed NMR studies revealed that in case of the ATH of *N*‐alkylimines, several different complexation modes occur when using CPAs, meanwhile only the (*E*) and (*Z*) binary complexes are formed in those reactions relying of DSI‐catalysis, which explains the superiority of the latter catalyst class for these transformations.^[39]^

**Scheme 13:**
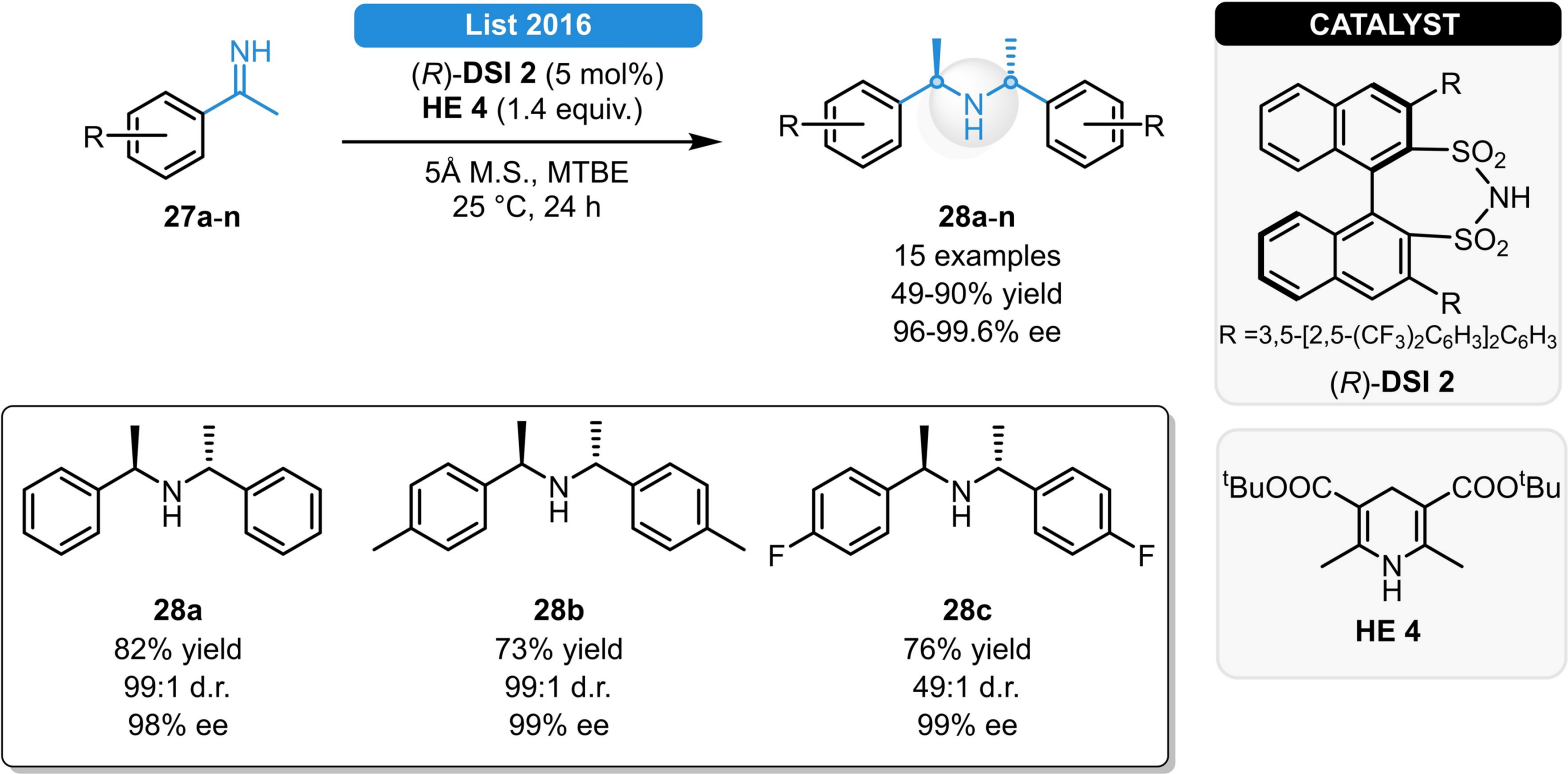
Chiral DSI‐catalyzed asymmetric reductive condensation of *N*−H imines.

Relying on chiral DSI‐catalysis, the List group reported the synthesis of C_2_‐symmetric secondary amines *via* reductive condensation of N−H imines (Scheme 13). Using the acid (*R*)‐**DSI 2** and Hantzsch ester **HE 4**, a series of optically active aromatic C_2_‐symmetric secondary amines (**28 a**–**n**) were prepared in moderate to good yields and in excellent stereocontrol. Without desiccant, partial hydrolysis of the products was observed resulting in inferior reactivity, indicating the important role of the molecular sieve in trapping the ammonia byproduct.^[40]^

Reductive amination provides another strategy for the synthesis of secondary or tertiary amines. It was already demonstrated by MacMillan and List in 2006, that the reduction of *in situ* formed imines can be achieved in a highly enantioselective fashion both for aldehyde and ketone substrates.[^31^, ^41^]

**Scheme 14:**
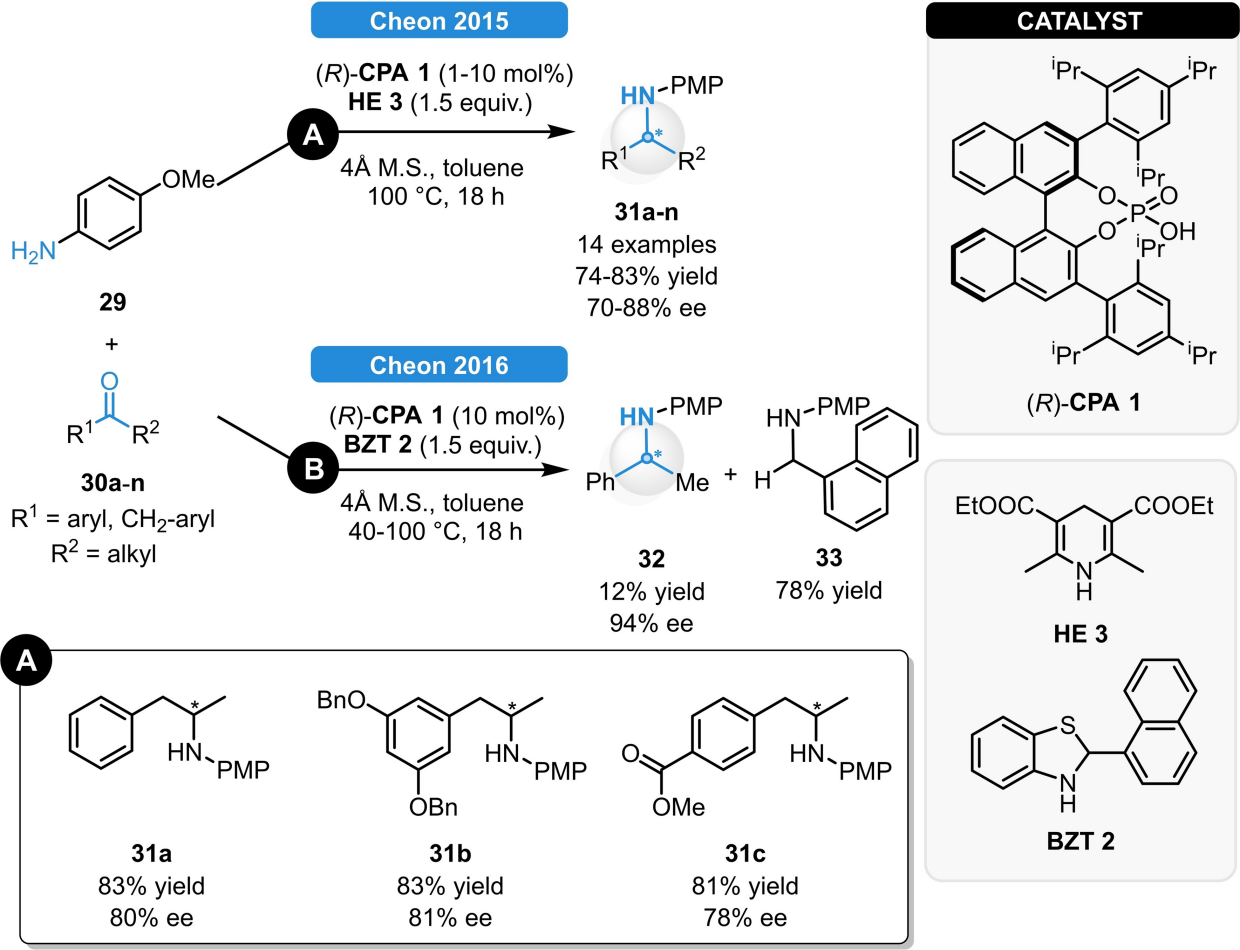
Synthesis of β‐aryl amines *via* reductive amination, relying on Hantzsch ester (**A**) or benzothiazoline reductants (**B**).

In 2015, Cheon *et al*. presented a new method for the synthesis of β‐aryl amines (Scheme 14, **A**). The reductive amination of various benzyl methyl ketones bearing substituents with different steric‐ and electronic properties (**30 a**–**n**) could be converted to the desired *N*‐PMP β‐aryl amines (**31 a**–**n**) in good yields and 70–88 % ee.^[42]^ Furthermore, gram‐scale experiments could be carried out as well using only 1 mol% (*R*)‐**CPA 1** without any decrease of the catalytic performance. Later on, the same group investigated the same reaction relying on benzothiazoline reductants (Scheme 14, **B**).^[43]^ While Hantzsch esters provided the corresponding chiral amines **31 a**–**n** exclusively; variable ratios of chiral and achiral product (e. g.: **32 a** and **33**) were obtained when using benzothiazolines as hydrogen donors. This unexpected phenomenon could be explained with the vulnerability of the reductant to *p*‐anisidine. As a competing pathway to the desired ketimine generation, this results in the formation of an aldimine species, which is then reduced with the remaining **BZT 2** to the achiral amine **33** (Scheme 14, **B**).

**Scheme 15:**
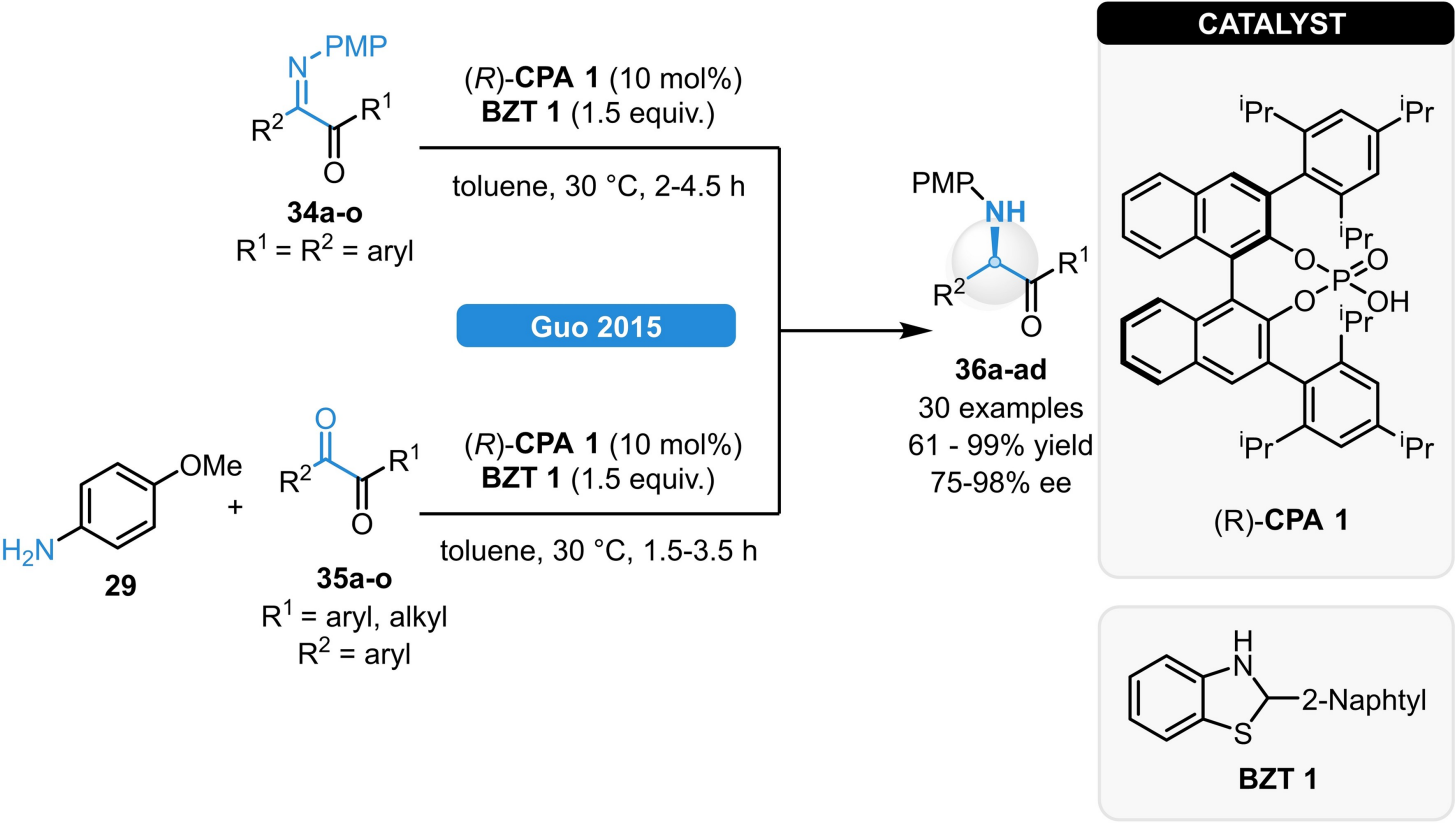
Strategies for the synthesis of α‐amino ketones *via* ATH and reductive amination.

The group of Guo reported the synthesis of optically active α‐amino ketones *via* direct ATH of imines and reductive amination of diketones, respectively (Scheme 15).^[44]^ Using (*R*)‐**CPA 1** together with the sterically demanding benzothiazoline **BZT 1**, a wide range of substrates could be used for both reaction types, providing a straightforward access to α‐amino ketones (**36 a**–**ad**) in 61–99 % yield and in 75–98 % ee. Gram‐scale experiments could be carried out with decreased phosphoric acid and Hantzsch ester loadings of 2 mol% and 1.2 equivalent; respectively, without any loss of the catalytic performance.

**Scheme 16:**
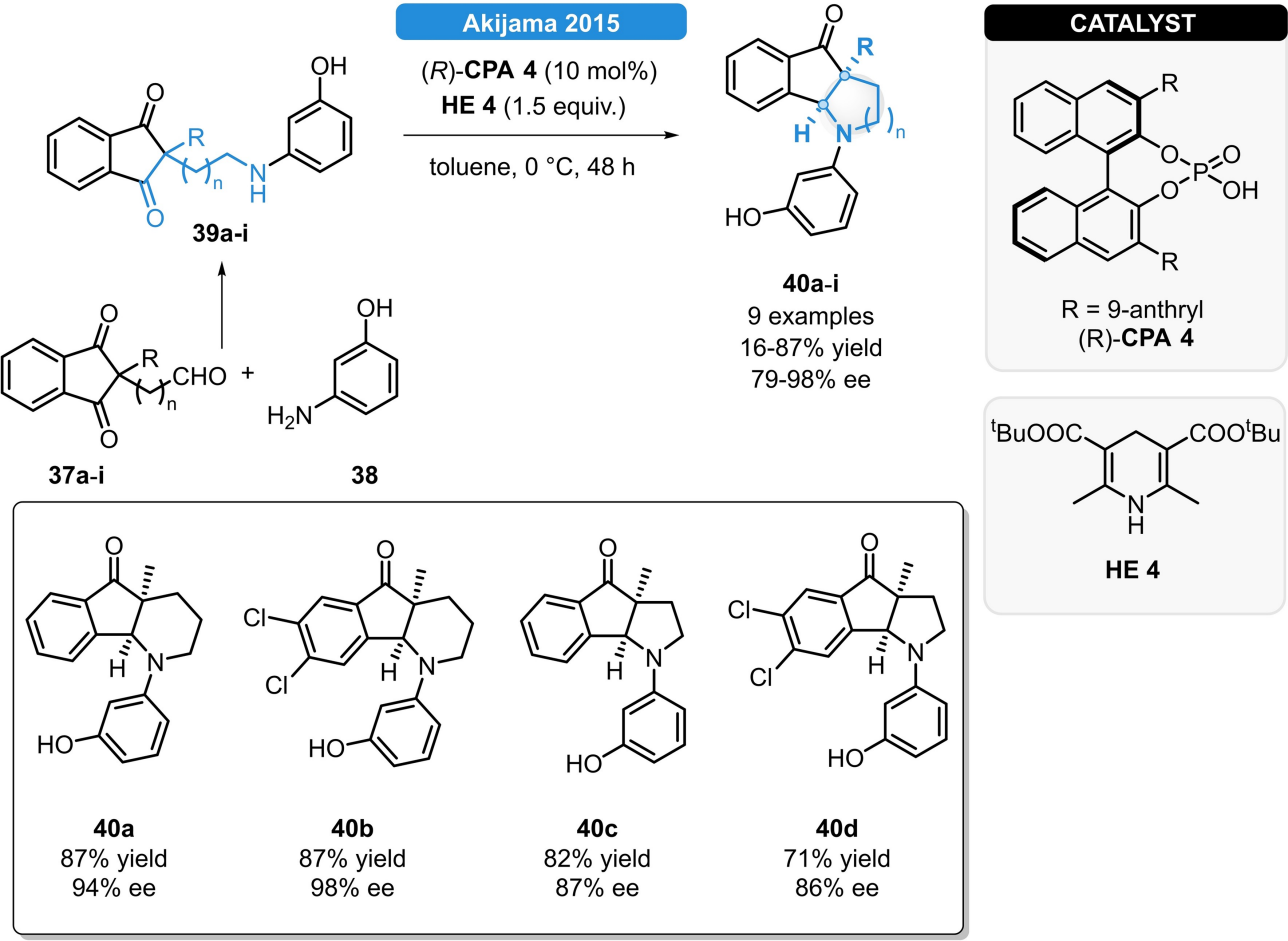
Synthesis of polyheterocycles *via* reductive amination.

In 2015, the Akiyama group reported the synthesis of fused piperidine and pyrrolidine‐based polyheterocycles *via* desymmetrization‐type reductive amination (Scheme 16): The indandione‐derived aldehyde **37 a**–**i** was reacted with *m*‐hydroxyaniline (**38**), followed by CPA‐catalyzed reduction. The 9‐anthracenyl‐substituted (*R*)‐**CPA 4**, together with the Hantzsch ester **HE 4** provided excellent enantioselectivities for adducts featuring 5–6 and 5–5 fused skeleton. Significantly worse results were obtained using *m*‐anisidine, suggesting that the reaction proceed through a cyclic transition state in which the (*R*)‐**CPA 4** activates both the aldehyde and the amine sources *via* hydrogen bonding.^[45]^

**Scheme 17:**
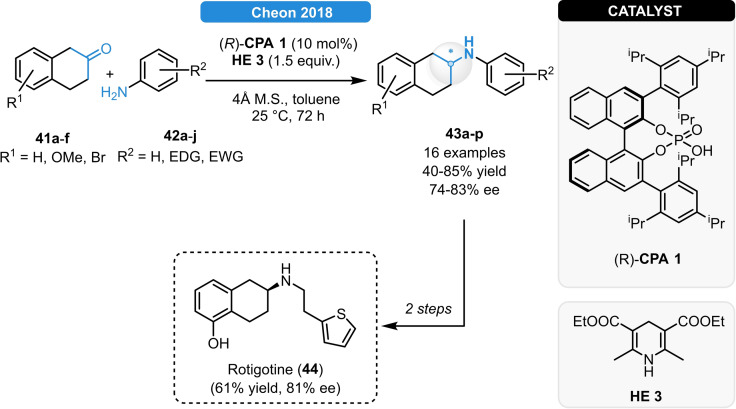
Reductive amination of β‐tetralones.

As the first example on the reductive amination of cyclic ketones, Cheon *et al*. disclosed the CPA‐catalyzed synthesis of β‐aminotetralins (Scheme 17).^[46]^ Various β‐tetralones (**41 a**–**f**) and anilines (**42 a**–**j**) bearing EWG or EDG groups could be readily reacted and the corresponding β‐aminotetralins (**43 a**–**p**) were obtained in moderate to good yields and in enantioselectivities up to 83 % ee; moreover, the synthetic utility was demonstrated by the enantioselective synthesis of the dopamine agonist drug Rotigotine (**44**).

Using 1‐hydrosilatrane (**3**), a novel hydrogen source was introduced for the reductive amination of ketones by Adler *et al*. Even though high yields and up to 84 % ee could be achieved, a rather high phosphoric acid loading of 30 mol% was required.^[47]^

**Scheme 18:**
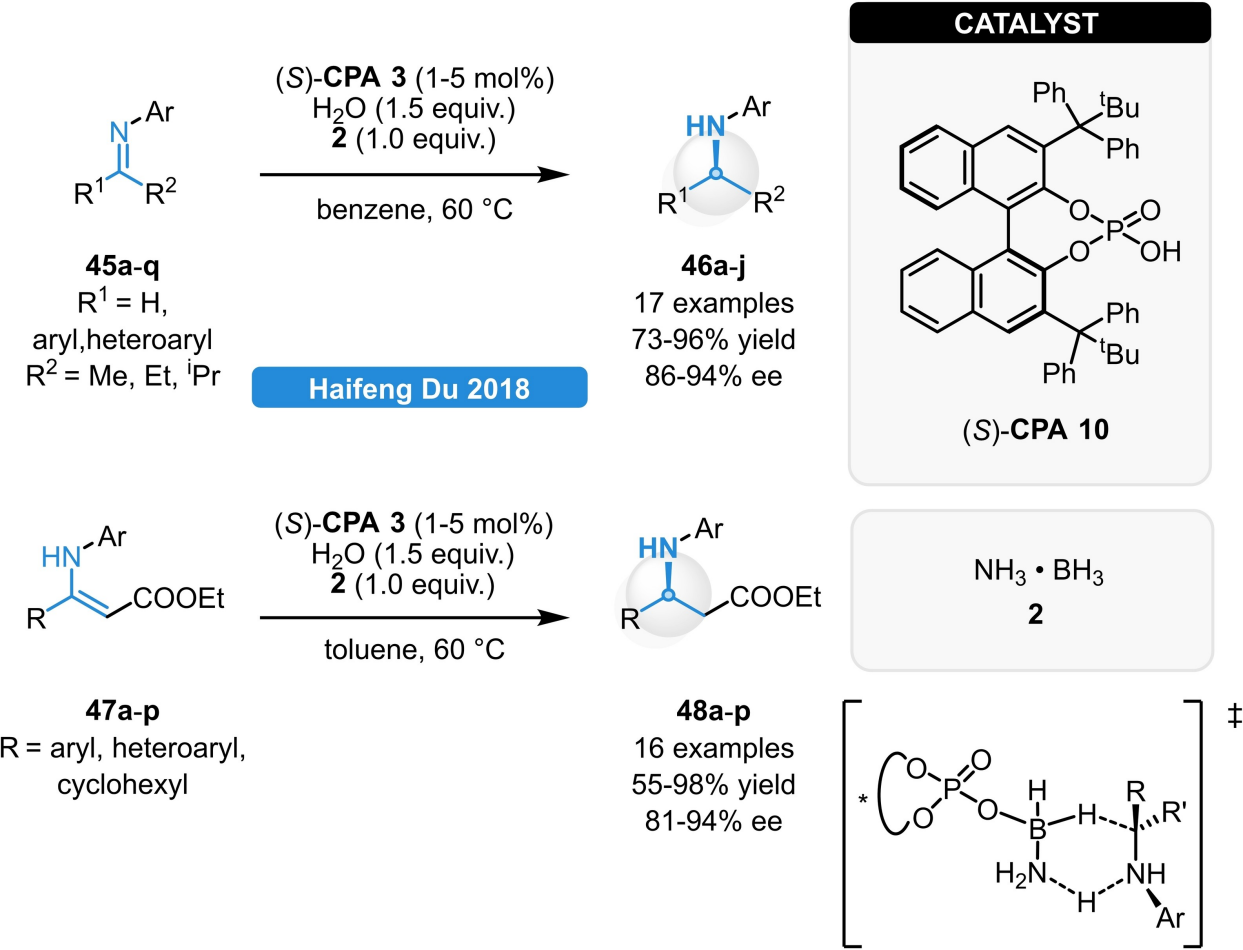
CPA‐catalyzed, ammonia borane‐mediated ATH of ketimines and β‐enamino esters.

The chiral ammonia borane composing of the (*S*)‐**CPA 10** and ammonia borane (**2**) was also successfully applied for the ATH of β‐enamino esters (**45 a**–**q**) and ketimines (**47 a**–**q**) with good yields and good to excellent stereocontrol (Scheme 18). It was proven, that the phosphoric acid acts as a simple Brønsted acid to generate the reactive chiral ammonia borane. Importantly, this species is continuously regenerated with the assistance of water and ammonia borane which also allows to lower the CPA loading below 1 mol%. The hydrogen transfer between the active species and the substrate is realized through a six‐membered transition state, as the transfer of the hydridic and protic hydrogens takes place in a simultaneous manner.^[48]^

## 4 Asymmetric Transfer Hydrogenation of N‐Heterocycles

**Figure 2:**
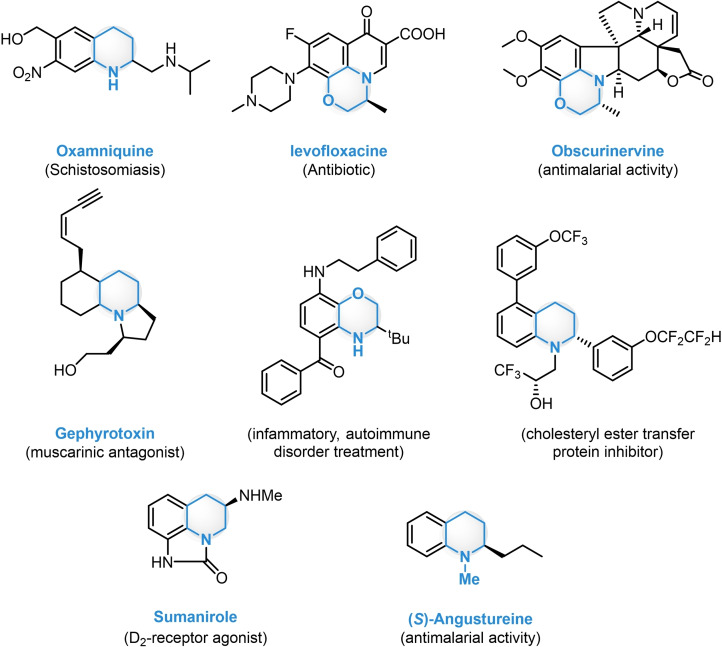
Various biological activity of saturated *N*‐heterocycles.

Enantioenriched (partially) saturated *N*‐heterocycles are undoubtedly crucial intermediates both for the agrochemical and pharmaceutical industries (Figure 2).[^49^, ^50^]

Since the pioneering works of Rueping on the field of chiral phosphoric acid catalyzed ATH of benzoxazines, benzoxazinones and benzothiazines,^[51]^ as well as on the dearomatization of quinolines,^[52]^ quinolones,^[53]^ quinoxalines,^[53]^ and pyridines,^[54]^ various methodologies were published for the asymmetric reduction of *N*‐heterocycles. This chapter aims to give an overview on recent advancements starting from 2014.

**Scheme 19:**
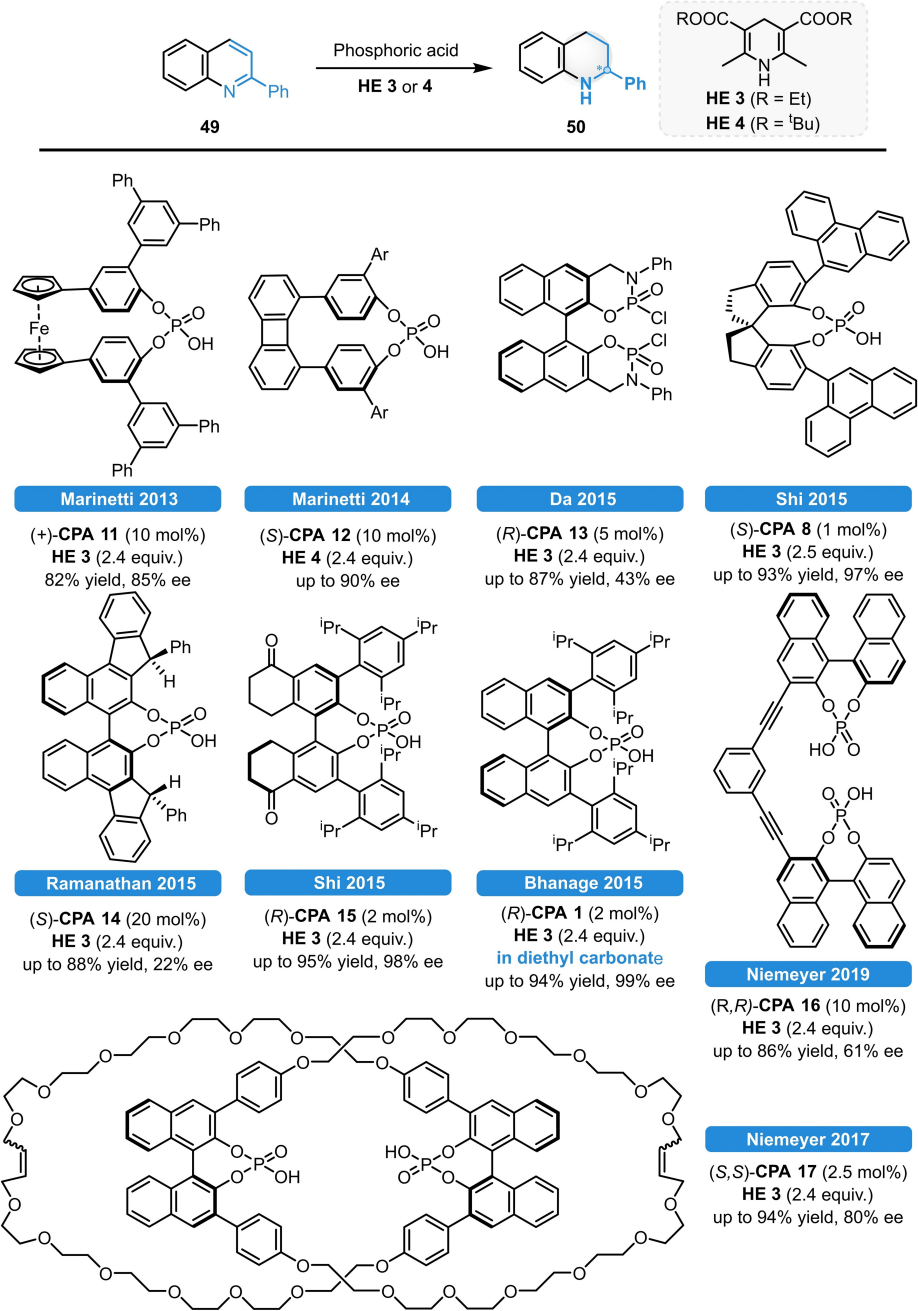
Recent advances for the ATH of **49**.

Serving as a benchmark reactions, various procedures were reported for the asymmetric reduction of 2‐phenylquinoline (**49**), relying on classical CPA catalysis in alternative reaction media,^[55]^ bitetralone‐modified CPAs,^[56]^ cyclophane‐ and cathenane‐based CPAs,[^57^, ^58^, ^59^] bisphosphoric acids,[^60^, ^61^] as well as using BIFOL‐^[62]^ and SPINOL‐derivatives (Scheme 19).^[63]^

**Scheme 20:**
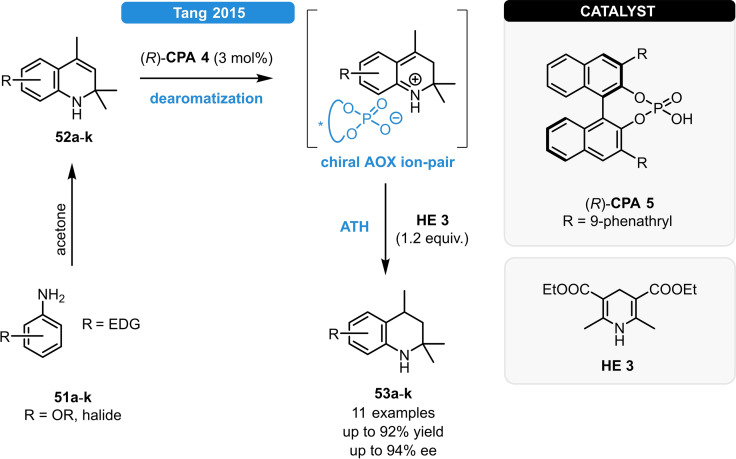
Formation of tetrahydroquinolines *via* AOX‐formation.

Tang *et al*. explored a novel, AOX‐mediated (AOX: aza‐*o*‐xylene) methodology to access dihydroquinolines (Scheme 20).^[64]^ In the presence of a Brønsted acid, the 1,2‐dihydroquinoline substrate (**52**) undergoes dearomatization forming a reactive and highly electrophilic aza‐*o*‐xylene (AOX) intermediate which then readily reacts with the **HE 3** reductant. After optimization, a series of 2,2,4‐trimethyltetrahydroquinolines (**53 a**–**k**) were obtained in high yields and in high to excellent enantioselectivities. This method provides not just a mild and metal‐free AOX‐formation, but it is also beneficial because of the rather simple substrate synthesis.

**Scheme 21:**
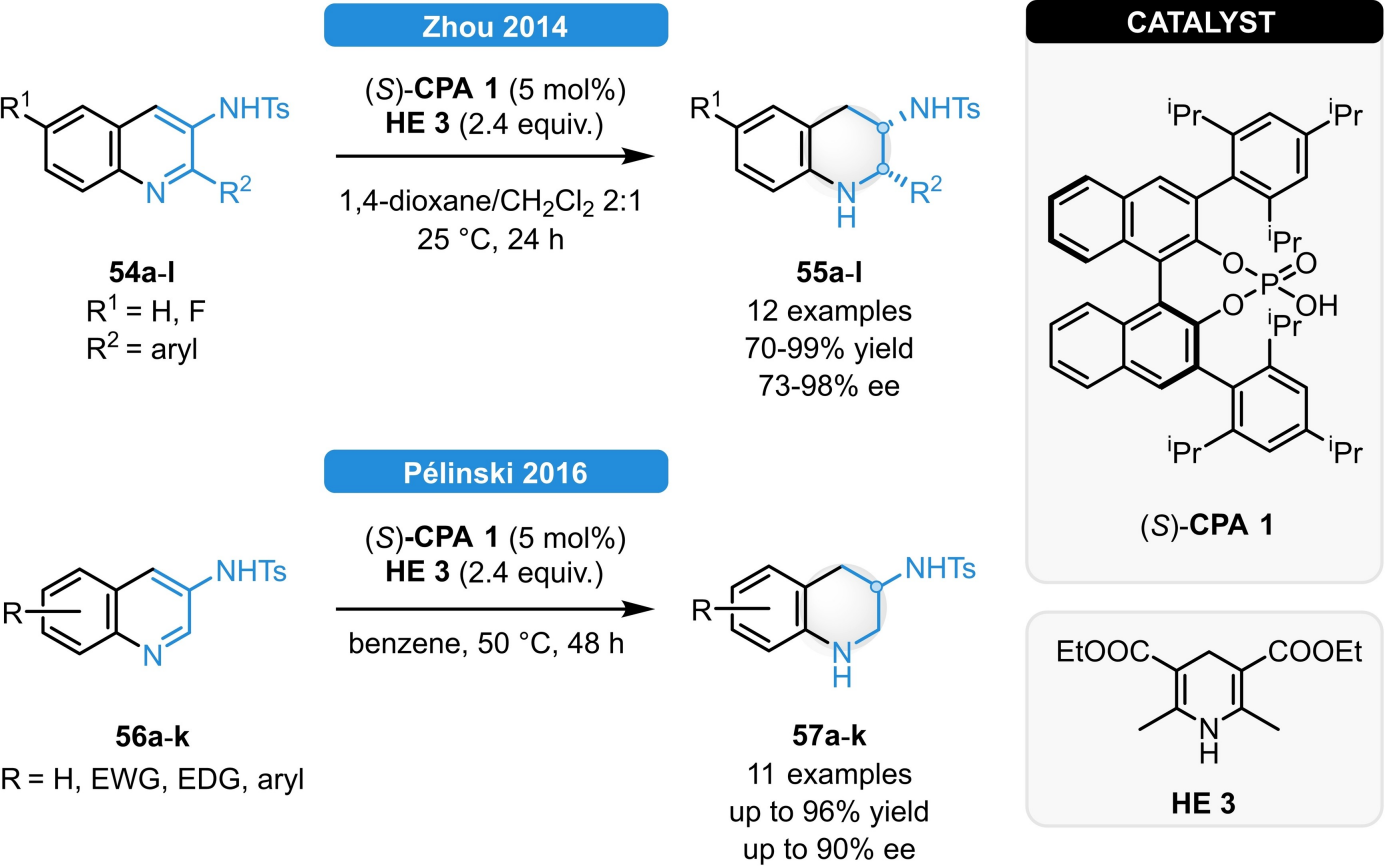
ATH of quinoline‐3‐amines.

In 2014, Zhou an co‐workers reported the ATH of 2‐arylquinoline‐3‐amines (Scheme 21, top).^[65]^ Even though no reaction was observed when using unprotected or phthaloyl‐protected amines, tosyl (Ts) or *tert*‐butoxycarbonyl (Boc) protected substrates could be smoothly converted to the corresponding tetrahydroquinolines. When using (*S*)‐**CPA 1**, a series of 2‐aryl substituted quinolines (**54 a**–**l**) could be reduced in good yields, excellent *cis* ‐diastereoselectivity and in 73–98 % ee. Importantly, the Ts‐group could be easily removed without loss of optical purity. As a result of isotopic labelling, no deuterium could be found at position C2, indicating that the reaction proceeds *via* an endocyclic imine intermediate and dynamic kinetic resolution is also involved for achieving high stereocontol. In analogy to this, the group of Pélinski showed that quinoline‐3‐amines featuring no C2‐substituents (**56 a**–**k**) can be successfully reduced in good yields and moderate to excellent ee as well; however, the protection of the amine functionality was found to be crucial for achieving high reactivity and enantioselectivity (Scheme 21, bottom).^[66]^

**Scheme 22:**
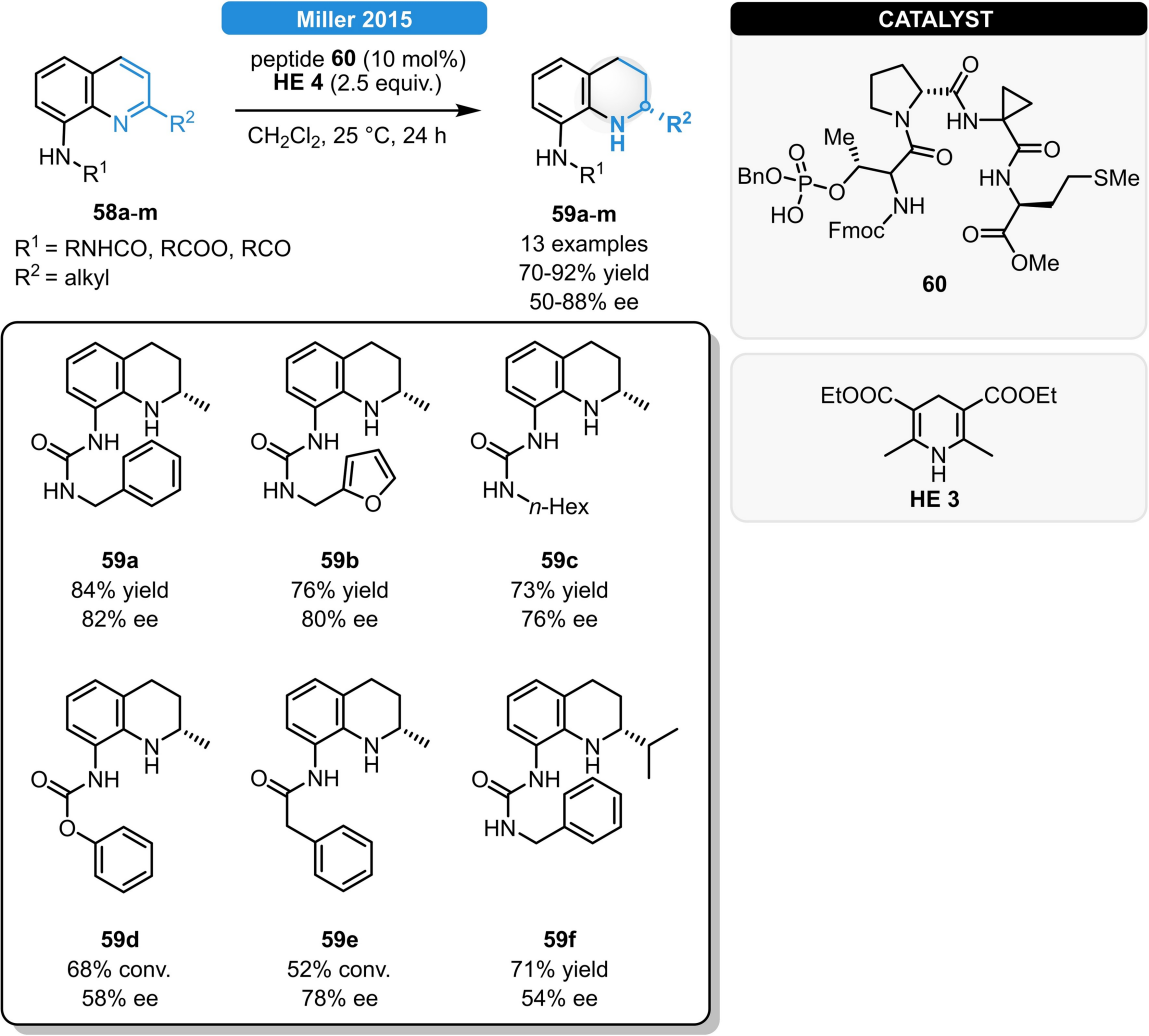
pThr‐containing peptides with CPA scaffold for the ATH of 8‐aminoquinolines.

Miller *et al*. constructed phosphothreonine (pThr)‐containing peptides which could be successfully used as novel CPA scaffolds. In total, 11 different tetrapeptides were screened for the ATH of 8‐aminoquinolines and after structural optimization, comparable results to those with (*R*)‐**CPA 1** were observed when using the peptide **60**. Various *N*‐protected substrates including aromatic and aliphatic ureas, carbamates and amides (**58 a**–**m**) were well tolerated, resulting in 70–92 % yield and in 50–88 % ee (Scheme 22). Even though such frameworks lack the *C*_2_‐symmetry and they also feature numerous rotatable bonds, the still rather high level of asymmetric induction could be rationalized through strong hydrogen bonding interactions between the substrate and the β‐turn of the peptide catalyst.^[67]^

**Scheme 23:**
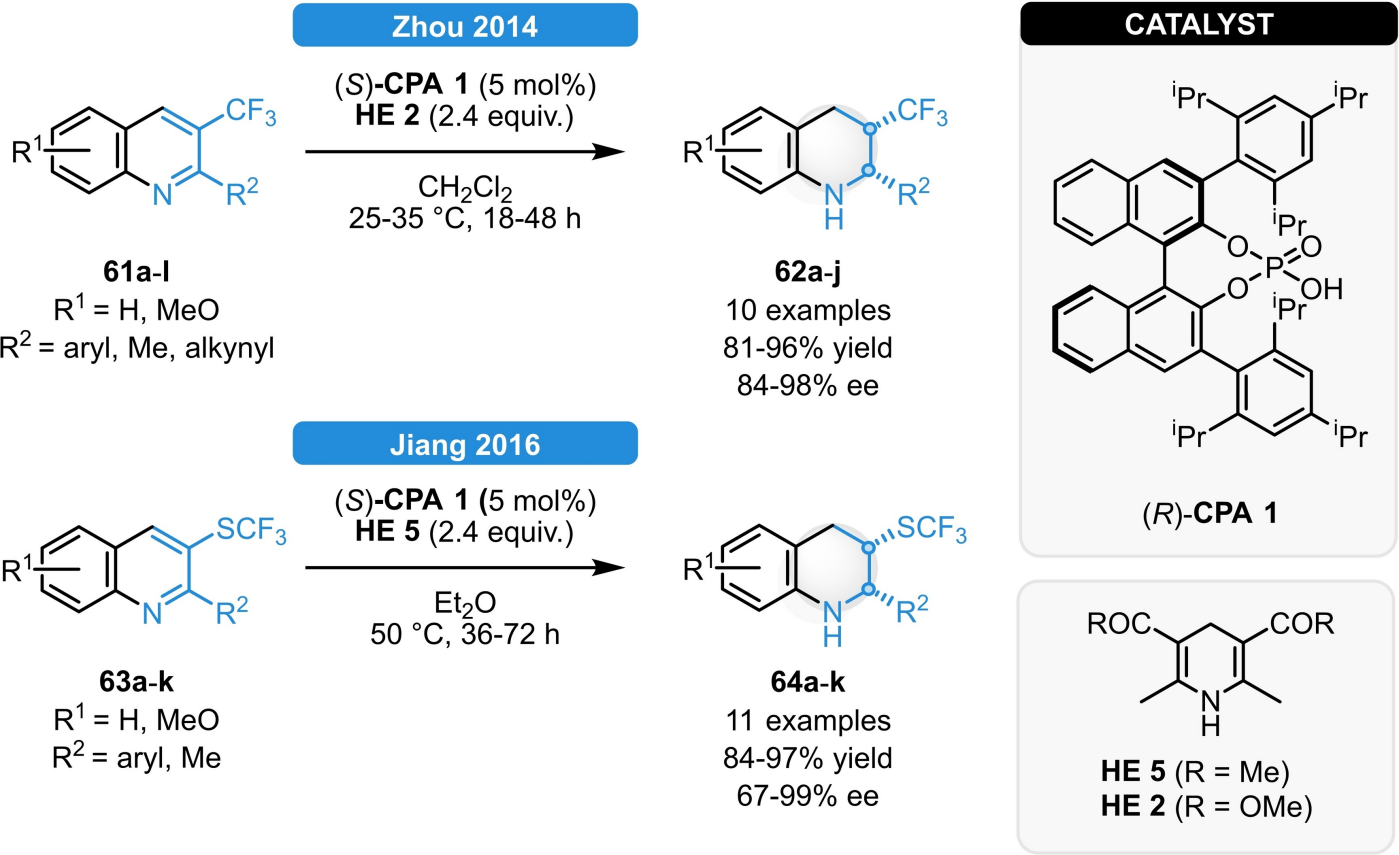
ATH of 3‐(trifluoromethyl)quinolines (top) and 3‐(trifluoromethyl‐thio)quinolines (bottom).

Zhou and co‐workers investigated the asymmetric reduction of 3‐(trifluoromethyl)quinolines.^[68]^ A series of 2‐arylsubstituted substrates (**61 a**–**l**) could be reduced in excellent yield and in 84–98 % ee. The presence of an aryl group in the C2‐position was found to be crucial for high diastereoselectivity: when using 2‐methyl or 2‐alkynyl substituents, the corresponding products were obtained in high enantioselectivity; albeit with poor diastereocontrol (Scheme 23, top). Under similar conditions, the ATH of 3‐(trifluoromethyl‐thio)quinolines (**63 a**–**l**) was achieved by the Jiang group (Scheme 23, bottom).^[69]^ Tetrahydroquinolines with C2‐aryl substituents (**64 a**–**l**) were formed in high yield and excellent diastereo‐ and enantioselectivity; meanwhile, the C2‐methyl analogue was obtained with inferior d.r. and ee values.

**Scheme 24:**
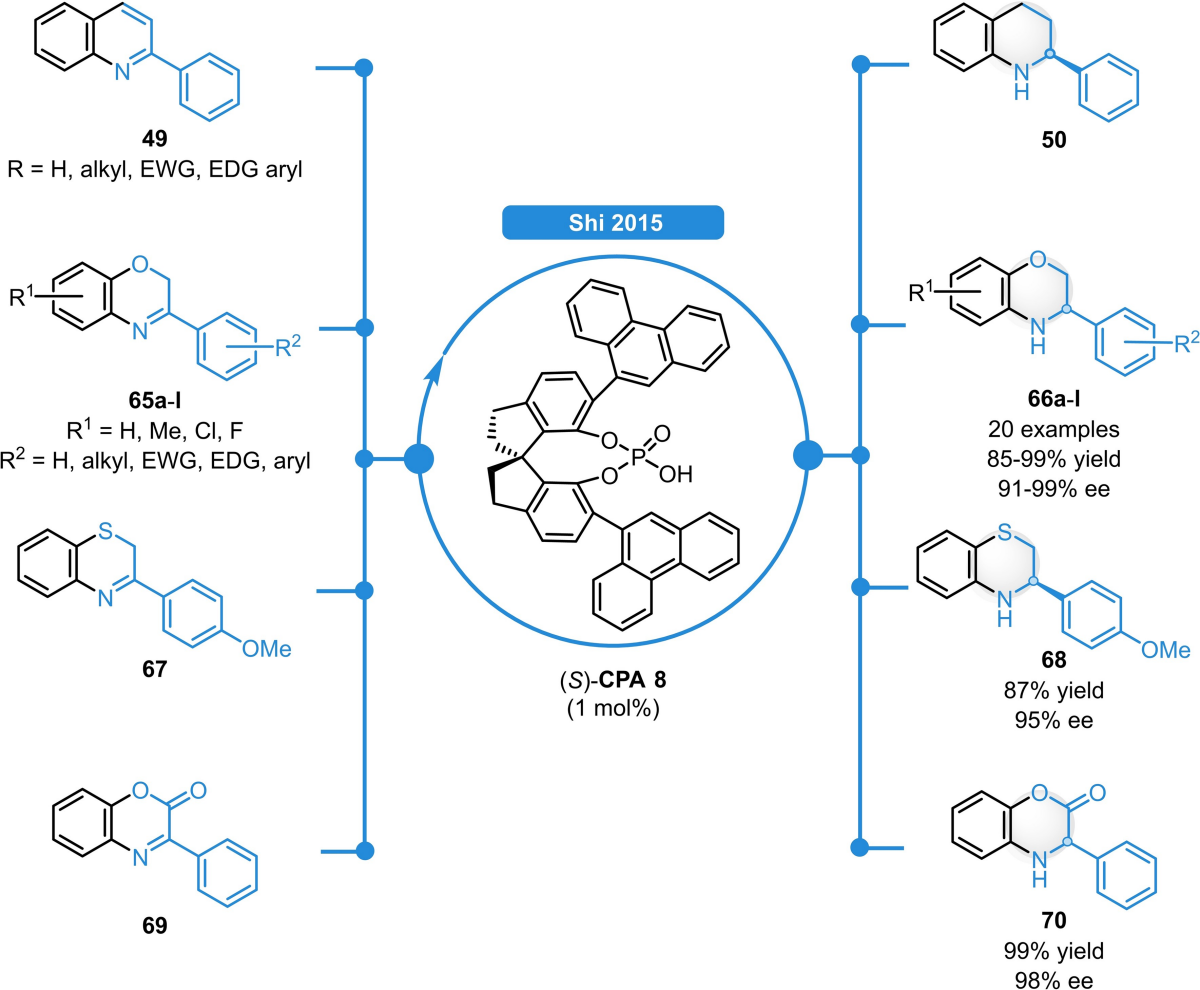
ATH of various *N*‐heterocycles catalyzed by SPINOL‐derived acid (*S*)‐**CPA 8**.

In 2015, Shi *et al*. successfully applied SPINOL‐derived phosphoric acids for the ATH of 1,4‐benzoxazines.^[63]^ Using (*S*)‐**CPA 8**, a broad range of 2‐aryl substituted substrates (**65 a**–**l**) could be reduced in 85–99 % yield and in excellent enantioselectivities (91–>99 % ee) with very low catalyst loading (Scheme 24). Notably, this method was also suitable for the ATH of diverse *N*‐heterocycles including quinolines (**49**), 1,4‐benzothiazolines (**67**), and benzoxazinanones (**69**) resulting in similarly high catalytic efficiency for all substrate classes.

**Scheme 25:**
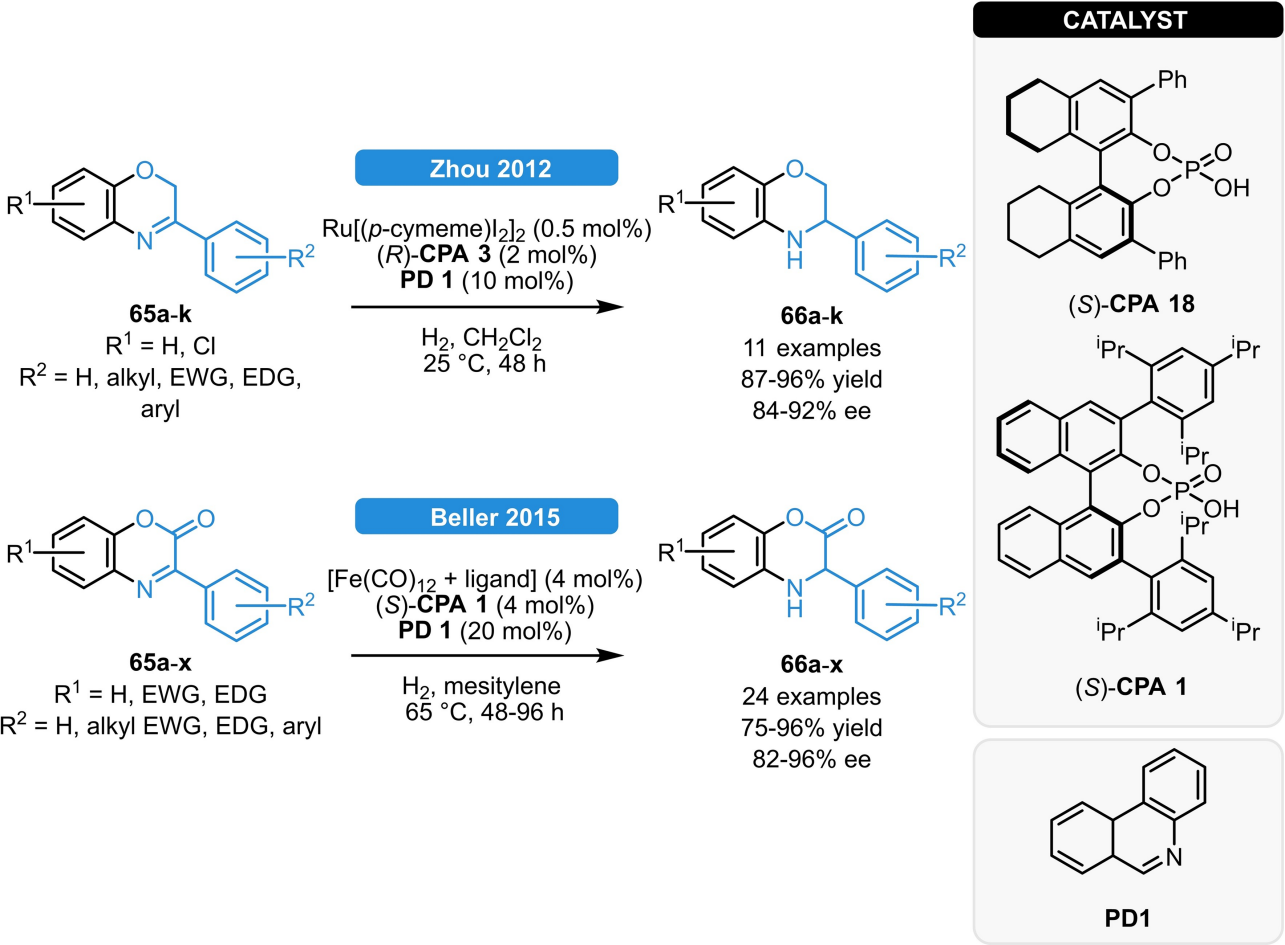
ATH of *N*‐heterocycles *via* Ru/CPA catalysis.

In analogy to their Ru/CPA‐catalyzed asymmetric transfer hydrogenation of imines (Scheme 11), the group of Zhou applied the same strategy for the ATH of 1,4‐benzoxazines, benzoxazinanones and related *N*‐heterocycles (Scheme 25, top),[^70^, ^71^] while Beller and co‐workers reported a relay Fe/CPA‐catalyzed asymmetric transfer hydrogenation of 1,4‐benzoxazinanones (Scheme 25, bottom).^[72]^ Both approaches relied on the use of sub‐stochiometric amount of phenanthridine biomimetic reductant in the presence of H_2_ as terminal reductant.

**Scheme 26:**
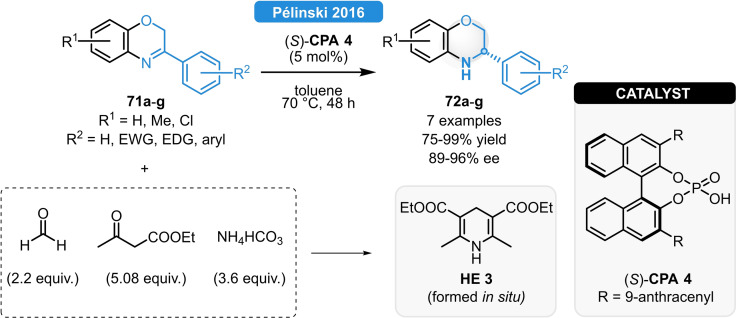
ATH of 1,4‐benzoxazines with *in situ* formed Hantzsch esters.^[74]^

Following the pioneering work of Zhou relying on the elegant combination of transition‐metal‐ and organocatalysis,^[73]^ Pélinski *et al*. reported a new, purely organocatalytic strategy for the ATH of 1,4‐benzoxazines relying on *in situ* formed dihydropyridine hydrogen sources (Scheme 26).^[74]^ The multicomponent reaction of NH_4_HCO_3_, formaldehyde and ethyl acetoacetate led to the *in situ* generation of **HE 3** and the substrates **71 a**–**g** could be reduced in 91–99 % yield and in 89–96 % ee. Even though such a nonpolar reaction medium is generally not suitable for the synthesis of Hantzsch esters, its immediate consumption in the subsequent ATH reaction could readily shift the equilibrium, ensuring high reactivity.

**Scheme 27:**
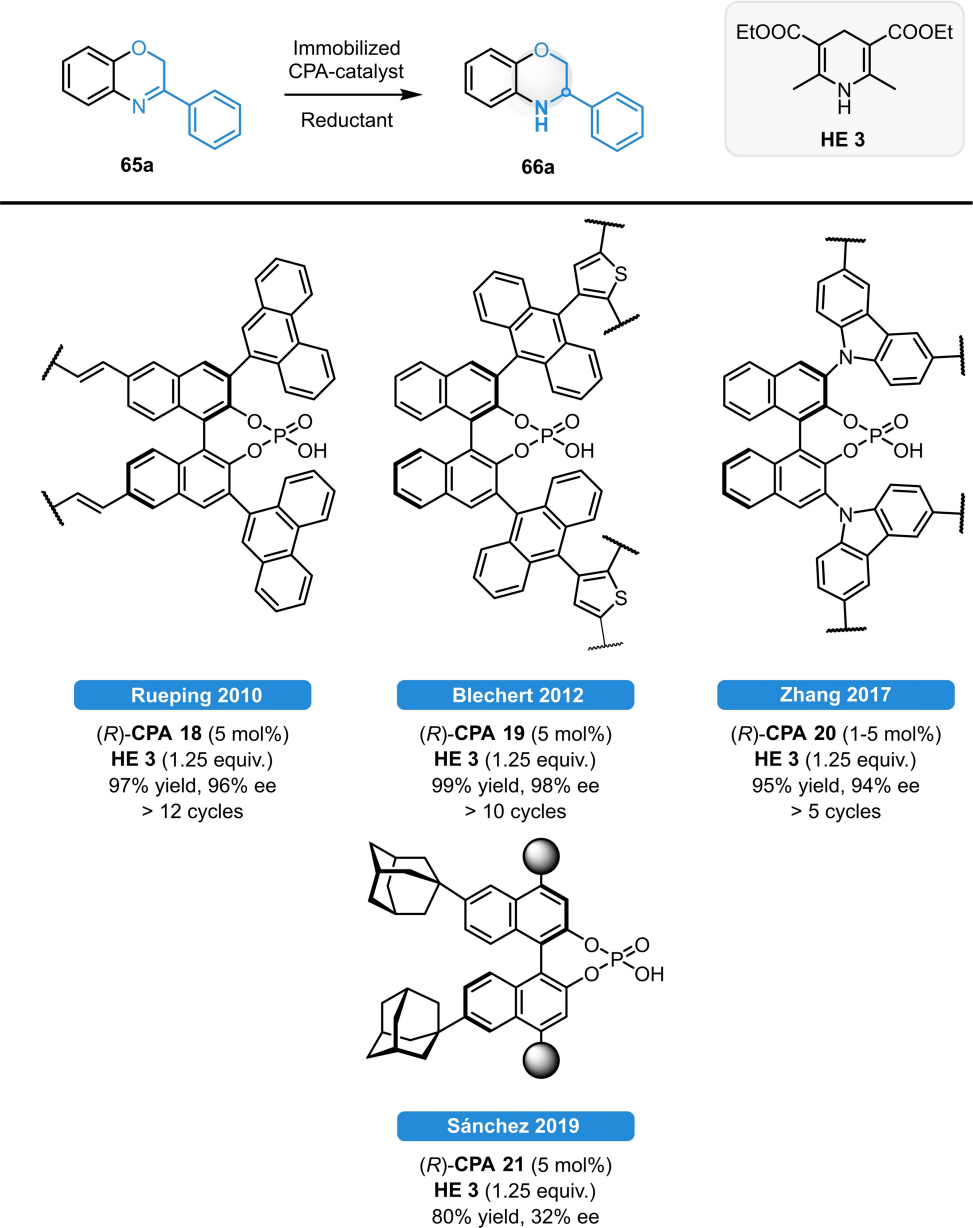
Catalyst immobilization strategies for the ATH of 1,4‐benzoxazines.

In addition to the asymmetric reductions in homogenous phase, various catalyst immobilization strategies were also developed for the ATH of 1,4‐benzoxazine derivatives (Scheme 27).^[75]^ In 2010, Rueping *et al*. reported the first immobilized chiral CPAs, as they successfully developed a series of polymer‐supported catalysts *via* radical polymerization of 7,7’‐styrene‐ or divinylbenzene‐linked CPA monomers. As the supported catalyst was made in form of small sticks, they could be used in an unusual „tea‐bag setup”: After completion of the reaction, the catalyst could be separated by simply pulling out the polymer stick from the reaction mixture. The catalyst (*R*)‐**CPA 18** provided excellent yield and enantioselectivity for the ATH of 1,4‐benzoxazine **65 a** and it could be used for at least 12 consecutive cycles. Blechert *et al*. prepared CPA‐derived microporous polymer networks *via* FeCl_3_‐mediated oxidative coupling of CPA monomers with 3‐(anthracen‐9‐yl)thiophene units (Scheme 27).^[76]^ The immobilized catalyst (*R*)‐**CPA 19** provided full conversion and 98 % ee for the ATH reaction of **65 a**; meanwhile, the catalyst could be reused for 10 cycles without any loss of reactivity and selectivity. Notably, the same catalyst was suitable for the ATH of 2‐aryl quinolines, but also for Friedel‐Crafts and Aza‐ene reactions. A similar approach was later presented by Zhang and co‐workers. Using carbazole‐substitution on the 3,3’‐position of the CPA they made a catalyst framework analogue to (*R*)‐**CPA 19**, resulting in high yields and enantioselectivities even when using only 1 mol% catalyst.^[77]^ Recently, the same reaction was investigated using adamantyl‐BINOL as platform for chiral porous polymer frameworks ((*R*)‐**CPA 21**). The product **66 a** could be isolated in good yield; however, only moderate enantioselectivity was observed.^[78]^ Even though they cannot be considered as pure organocatalysts, metal‐organic frameworks (MOFs) can also provide a nice alternative for the heterogenization of chiral Brønsted acids resulting in a highly porous catalyst framework and offering a high density of catalytic sites. Jiang *et al*. first described the synthesis of three different CPA‐based 3D In‐MOFs. They observed, that despite to their different structures, the CPAs are periodically aligned within the channels which makes the MOF framework catalytically active towards transfer hydrogenations of imines and benzoxazines.^[79]^ Very recently they extended their concept to non‐noble metal‐based structures. With the CPA‐based 3D Ca‐MOF they could achieve similarly high catalytic activity and enantioselectivities to those obtained with 3D Ir‐MOFs.^[80]^

**Scheme 28:**
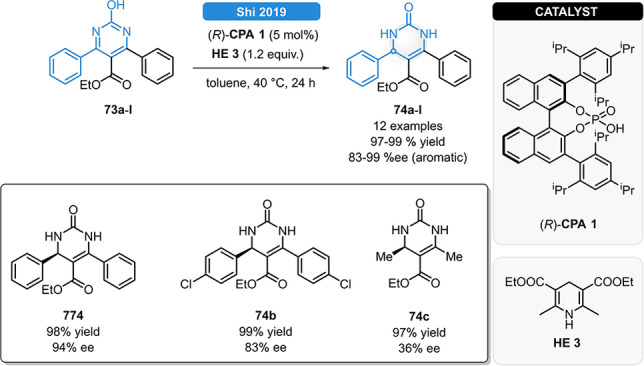
Synthesis of DHPDs.

As an alternative to the Biginelli reaction, Shi *et al*. described the synthesis of 3,4‐dihydropyrimidin‐2(1*H*)‐ones (DHPDs, **77 a**–**l**) *via* asymmetric reduction of pyridines (Scheme 28).^[81]^ Using the phosphoric acid (*R*)‐**CPA 1**, a small set of 3,4‐diarylpyridines (**73 a**–**l**) could be reduced in excellent yield and enantioselectivities. The high reactivity could be maintained for aliphatic substrates; however, lower ee values were observed.

**Scheme 29:**
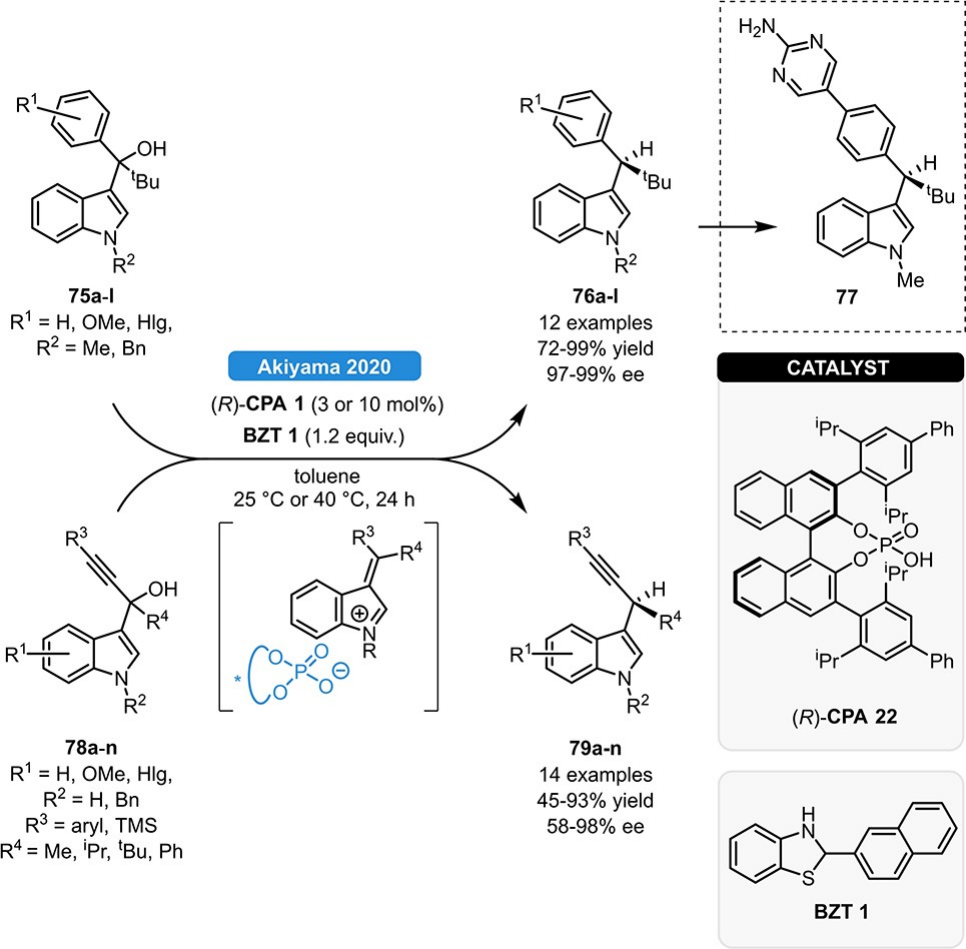
Dehydroxy‐hydrogenation of 3‐indolylmethanol derivatives.

Very recently, the group of Akiyama reported the asymmetric dehydroxy‐hydrogenation of 3‐indolylmethanols (Scheme 29).^[82]^ Relying on (*R*)‐**CPA 22** and **BZT 1**, variously substituted indolyl‐ (**75 a**–**l**) and indolyl propalgyl methanols (**78 a**–**n**) could be reduced in high yields and in >90 % ee; meanwhile substituents on the alkyne and on the arene moieties were both well tolerated. In order to demonstrate synthetic applicability, the product **76 a** was transformed to the leukotriene production inhibitor **77** *via* Suzuki‐Miyaura cross‐coupling without any loss of optical purity. The high enantioselectivity is originated from the tight ion‐pairing of the **CPA 22** with the dehydrated alkylideneindoleninium ion.

In the same year, Song *et al*. applied *in situ* formed CPA‐boron complexes for the direct ATH of C2‐aryl‐substituted, *N*‐unprotected indoles.^[83]^ The [H]_8_‐derived (*R*)‐**CPA 7** provided excellent enantioselectivities for a wide range of indole substrates. For achieving high enantioselectivities, low temperature and 3.0 equivalent of H_2_O were both necessary.

## 5 Organocatalytic Cascade and One‐Pot Reactions

Cascade and one‐pot reactions are extreme versatile tools for the rapid construction of high molecular complexity. The overall reaction can be considered as a sequence of several reaction steps that either take place simultaneously or one after the other. As these reaction steps proceed in a single operation under identical/very similar reaction conditions without the necessity of isolating the reaction intermediates, such transformations provide a highly step‐ and atom economic alternative to classical multi‐step synthetic strategies. Allowing selective and well distinct modes of substrate activations, organocatalysts are particularly suitable for such kind of transformations. One of the first CPA‐ catalyzed organocatalytic cascade reaction was performed in the group of Rueping in 2008. The three‐component reaction of an enamine, a vinyl ketone and a Hantzsch ester proceeded *via* a six‐step cascade, all being catalyzed by the same phosphoric acid (*R*)‐**CPA 4**, providing an easy access to tetrahydropyridines and azadecalinones in a highly enantioselective fashion.^[84]^

**Scheme 30:**
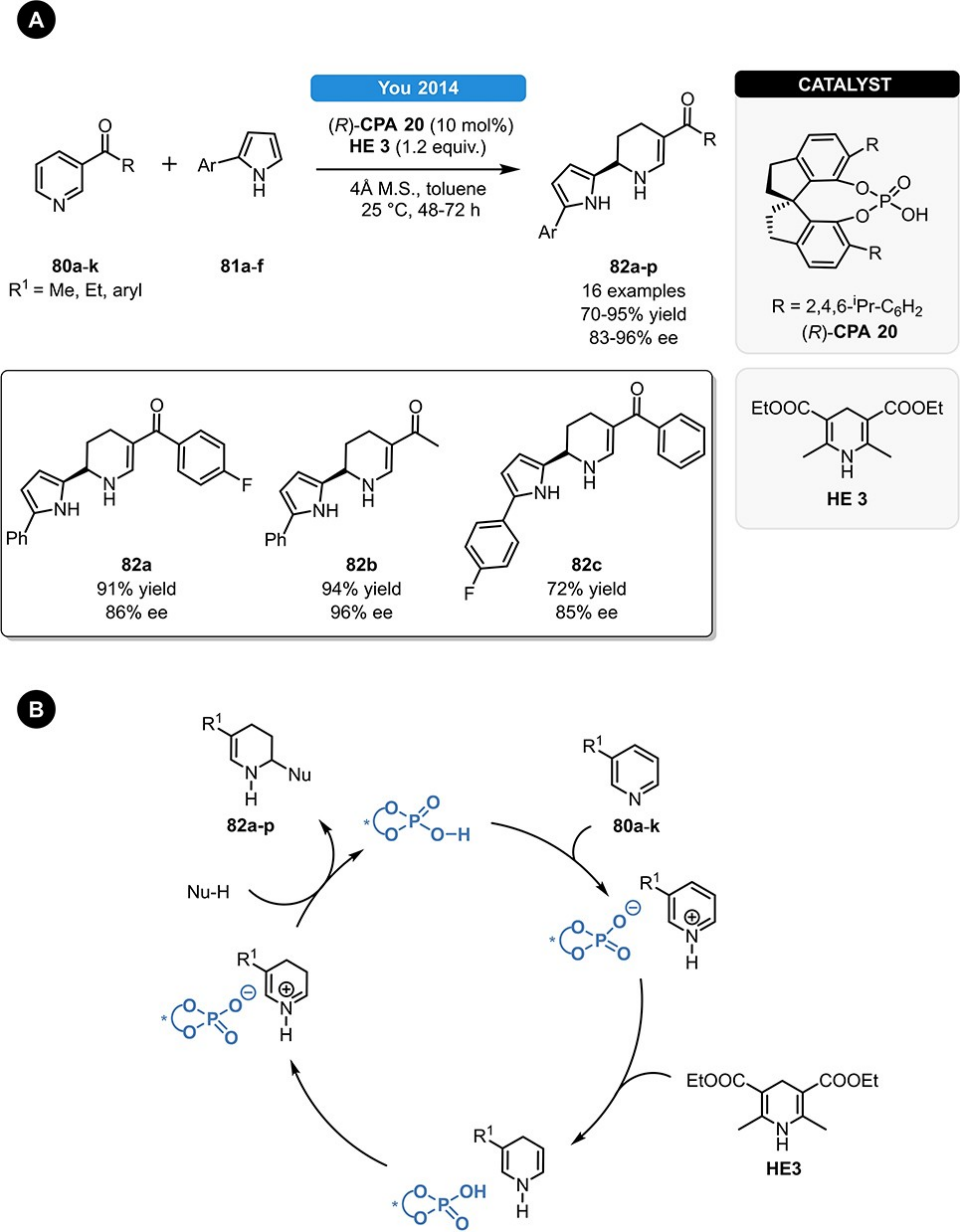
Synthesis of piperidines *via* asymmetric dearomatization/aza‐Friedel‐Crafts alkylation cascade reaction.

In 2014, You *et al*. reported an asymmetric dearomatization/aza‐Friedel‐Crafts alkylation cascade for the synthesis of substituted piperidines (Scheme 30, **A**).^[85]^ When using the SPINOL‐derived (*R*)‐**CPA 20**, various 3‐substituted pyridines (**80 a**–**k**) and 2‐arylpyrroles (**81 a**–**f**) could be readily reacted under mild reaction conditions, furnishing the highly functionalized piperidine **82 a**–**p** in high yields and in good to excellent enantioselectivity. The authors proposed that after the initial ATH reaction, the generated enamine isomerizes to the corresponding iminium form, which can react with the 2‐arylpyrrole nucleophiles (Scheme 30, **B**).

**Scheme 31:**
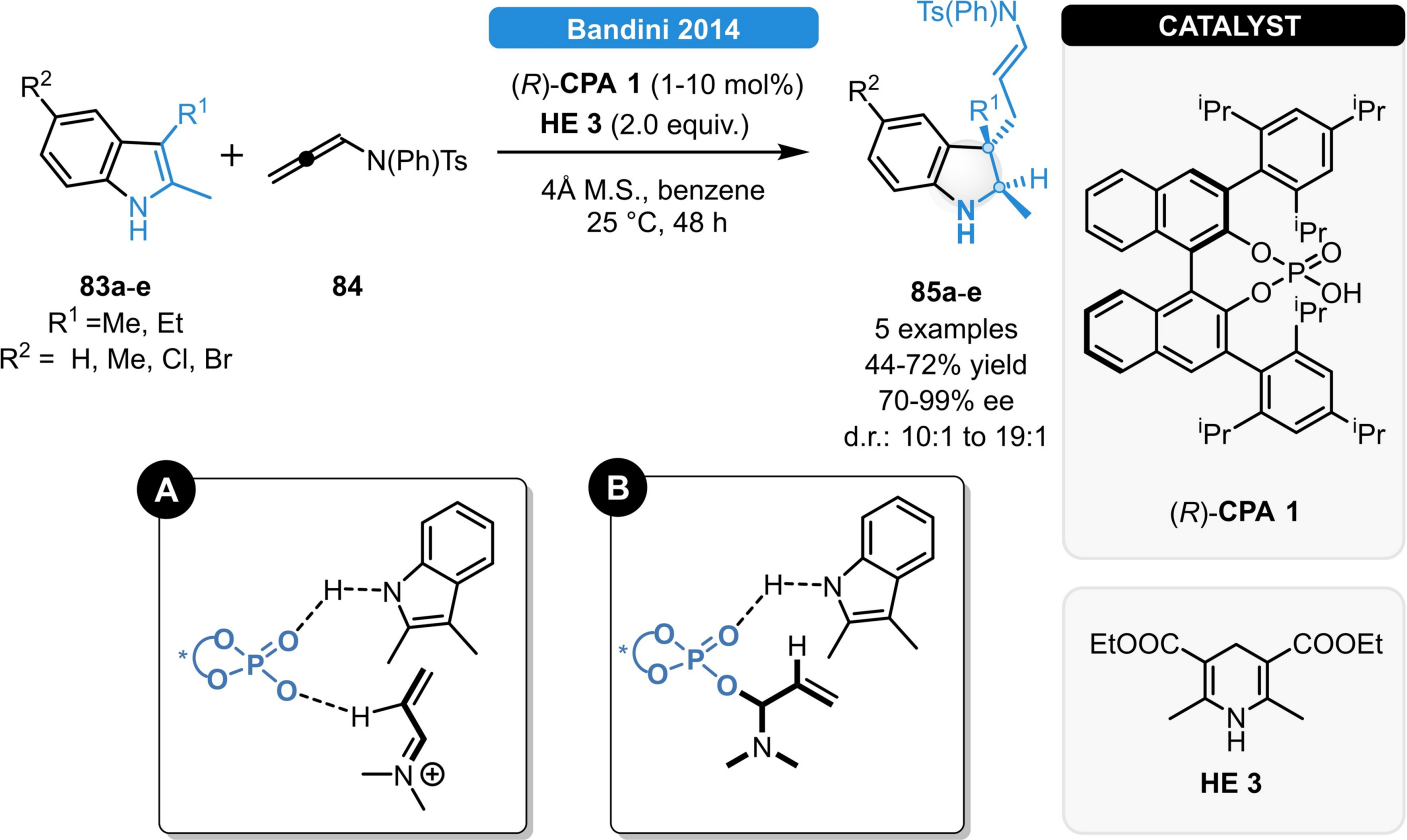
Dearomatization/ATH cascade for the synthesis of highly functionalized indole moieties (top) and the possible activation modes (bottom, **A** and **B**).

In the same year, Bandini *et al*. reported a metal‐free strategy for the synthesis of 3,3‐disubstituted indolines (Scheme 31). The (*R*)‐**CPA 1** as single catalyst could successfully promote a highly enantioselective dearomatization/transfer hydrogenation cascade *via* the electrophilic activation of allenamides. The condensation of 2,3‐disubstituted or 2,3,5‐trisubstituted indoles (**83 a**–**e**) with *N*‐aryl allenamide **84** resulted in the formation of indolenine intermediates, which were subsequently reduced *in‐situ* to the indoline products (**85 a**–**e**) in excellent diastereo‐ and enantioselectivity. It is believed that the selective protonation of the allenamide at the β‐position provides an α,β‐unsaturated iminium intermediate, and subsequent Michael‐addition and transfer hydrogenation affords the products. The (*R*)‐**CPA 1** acts as a bifunctional catalyst activating the allenamide **84** either *via* non‐covalent or covalent interactions (Scheme 31, **A** and **B**).^[86]^

**Scheme 32:**
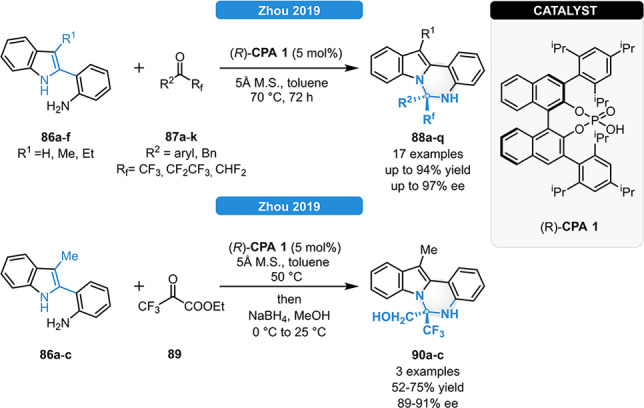
CPA‐mediated synthesis of 5,6‐dihydroindolo[1,2‐c]quinazolines (top) and its application for the preparation of α‐diamino acid derivatives (bottom).

In 2019, Zhou and co‐workers reported a CPA‐catalyzed condensation/amine addition cascade for the synthesis of 5,6‐dihydroindolo[1,2‐*c*]quinazolines (Scheme 32, top)^87^. Various 2‐(1*H*‐indolyl)anilines (**86 a**–**g**) and fluorinated ketones (**87 a**–**k**) could be readily reacted in the presence of 5 mol% (*R*)‐**CPA 1**. The CF_3_‐group was found to play a crucial role in the reaction: as a potent hydrogen‐bond acceptor, interactions either with the indole N−H or with the chiral phosphoric acid could result in higher enantiocontrol. When using the α‐ketoester **89** as reagent, the cascade reaction followed by subsequent reduction with NaBH_4_ provided an easy access to the highly enantioenriched α‐diamino acid derivatives **90 a**–**c** (Scheme 32, bottom).^[87]^

**Scheme 33:**
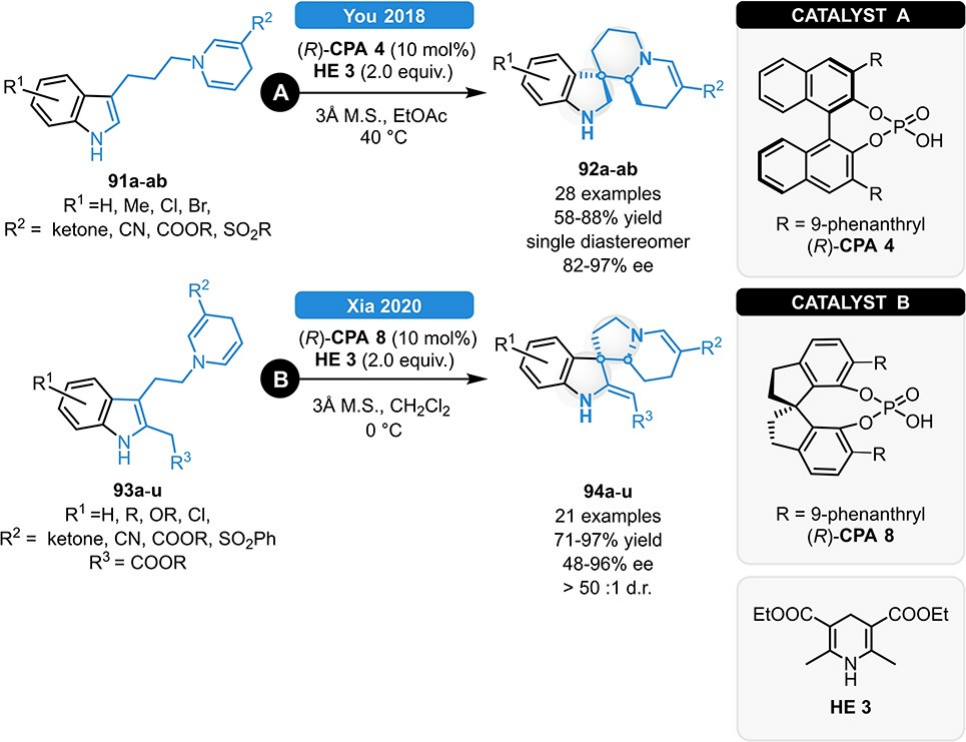
Different concepts for the synthesis of spiroindolines *via* CPA‐catalyzed cascade reactions.

In 2018, You and co‐workers realized the synthesis of spiroindolines *via* cascade isomerization/spirocyclization/transfer hydrogenation reaction (Scheme 33, **A**). Under optimized reaction conditions, a wide range of indolyl dihydropyridine substrates (**91 a**–**ab**) featuring various ketone moieties, esters, cyano or sulfonyl groups on the 1,4‐dihydropyridine sub‐unit were well tolerated, providing the corresponding spiroindoline **92 a**–**ab** in 58–88 % yields and in 82–97 % ee; moreover, EDG groups and halogen atoms in the R^1^ position were also compatible with the reaction. The reaction was found to be extremely diastereoselective, providing a single diastereomeric product in most of the cases.^[88]^ Recently, Xia and co‐workers reported a similar method for the synthesis of 2,7‐diazaspiro[4.4]nonane indolines (**94 a**–**u**, Scheme 33, **B**). Using the SPINOL‐derived (*R*)‐**CPA 8**, a comparably broad range of substrates could be applied resulting in excellent diastereoselectivity and 60–96 % ee. The nature of the R^2^ and R^3^‐groups was found to be crucial: electron‐poor and sterically demanding R^2^‐groups resulted in high enantioselectivity; meanwhile, the electron withdrawing R^3^‐ester unit facilitated the formation of the spiro‐products and inhibited the alternative Pictet‐Spengler pathway.^[89]^

**Scheme 34:**
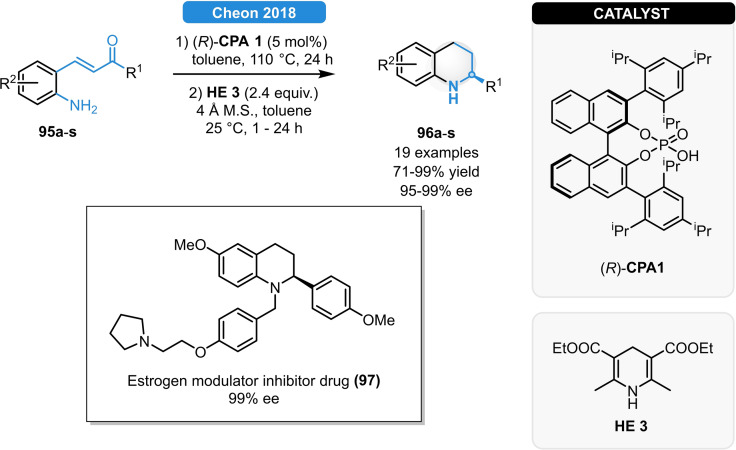
Synthesis of tetrahydroquinolines *via* dehydrative cyclization/ATH cascade reaction.

In 2018, the group of Cheol‐Hong Cheon managed to synthesize 2‐substituted tetrahydroquinolines in a highly enantioselective fashion by a dehydrative cyclization/ATH reaction sequence (Scheme 34).^[90]^ Unlike previous works, the two‐step, one‐pot transformation could be carried out by using a chiral phosphoric acid as sole catalyst. After identifying the (*R*)‐**CPA 1** as most suitable catalyst, a broad range of 2‐aminochalcones (**95 a**–**u**) could be converted to the corresponding 2‐substituted tetrahydroquinolines in uniformly high 94–99 % ee. Moreover, the strategy was applicable for the synthesis of the estrogen modulator inhibitor **97** in significantly higher yield and identical enantioselectivity than previous literature reports.

**Scheme 35:**
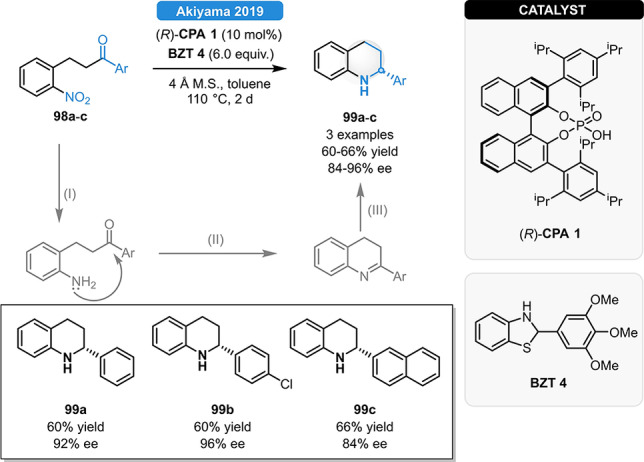
One‐pot synthesis of chiral quinolines *via* reduction of nitroarenes in a reduction/cyclization/reduction cascade.

Recently, the Akiyama group established a highly enantioselective methodology for the synthesis of 2‐aryltetrahydroquinolines (**99 a**–**c**).^[91]^ After optimizing the reduction of various achiral nitroarenes, a three‐step reaction cascade was developed: the initial reduction of the nitroarene (I) was followed by intramolecular cyclization (II) and asymmetric transfer hydrogenation (III). Relying on (*R*)‐**CPA 1** and **BZT 4** as a H‐donor, the corresponding products were obtained in 60–66 % yield and in 84–96 % ee (Scheme 35).

## 6 Conclusions

Organocatalytic asymmetric transfer hydrogenations relying on biomimetic hydrogen sources have proven to be very attractive and popular alternatives to classical transition‐metal‐based ATH reactions. The introduction of BINOL‐derived chiral phosphoric acids could basically revolutionize this field, providing straightforward protocols for the reduction of an extremely diverse pool of substrates featuring C=C, C=N and C=O double bonds. Apart from providing a safe, simple and metal‐free alternative for asymmetric hydrogenations, several advances could also successfully tackle the challenge of high catalyst loadings, relying on catalyst amounts similar to those used in transition‐metal catalysis. Apart from all these advantages, chiral phosphoric acid‐catalyzed ATH reactions are more and more often applied for cascade transformations as well as in total synthesis, indicating its high potential for various industrial applications. Given the juvenileness of this particular field of organocatalysis, a broad range of novel future applications is still expected.

## Conflict of interest

The authors declare no conflict of interest.

## Biographical Information

*Ádám Márk Pálvölgyi received his master's degree in organic chemistry in 2017 from the Budapest University of Technology and Economincs. In 2021, he obtained his PhD degree from the Technical University of Vienna (TU Wien) under the supervision of Prof. Katharina Schröder. He is currently a post‐doctoral researcher in the same group. His current research focuses on asymmetric organocatalysis and photocatalysis*.

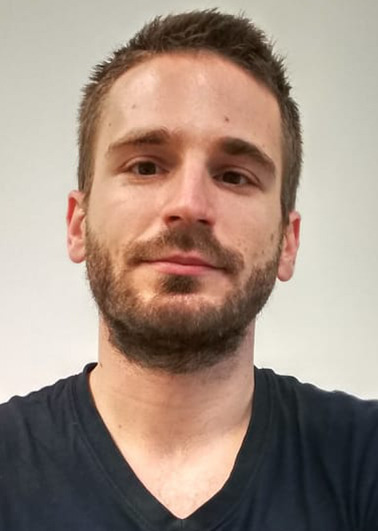

## Biographical Information

*Fabian Scharinger carried out his Master's degree in the group of Katharina Schröder in 2019 at the Technical University of Vienna under the co‐supervision of Ádám Márk Pálvölgyi. He then continued with his PhD in the same group under the supervision of Prof. Katharina Schröder, focusing on the development of novel organocatalysts and their application in asymmetric synthesis*.

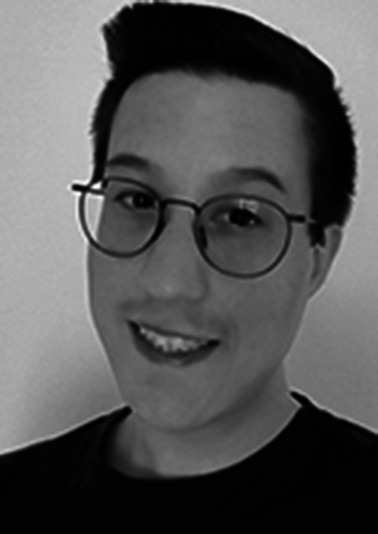

## Biographical Information

*Michael Schnürch has carried out his PhD thesis in the group of Prof. Peter Stanetty and received his PhD in 2005 from TU Wien. He was then Post‐Doc with Prof. Dalibor Sames at the Columbia University in New York City (as Erwin Schrödinger fellow) and conducted research in the field of decarbonylative coupling reactions and sp* ^*3*^ *C−H activation. After his return, he became Assistant Professor at TU Wien and completed his habilitation in 2013. He was promoted to privatdozent and in 2016 to Associate Professor for Organometallic Chemistry. Additionally, he was the chair of the COST Action CHAOS (C−H Activation in Organic Synthesis) and the European Symposium on Organic Chemistry (ESOC 2019) in Vienna. His research interests are located in the field of synthesis of heterocyclic compounds for the manipulation of cell differentiation and GABAA receptors, C−H activation of sp* ^*3*^ *centers, the substitution of gaseous reagents for solid alternatives, green chemistry, and flow chemistry*.

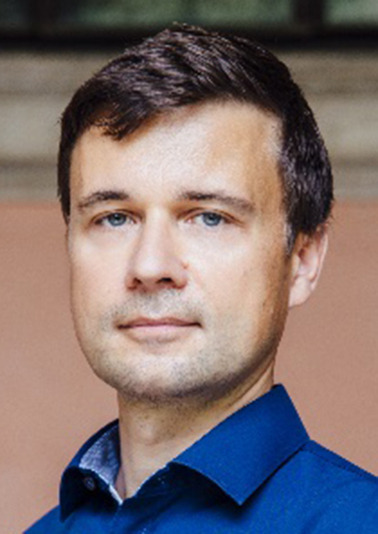

## Biographical Information

*Katharina Schröder (born K. Bica) received her PhD in Technical Chemistry from TU Wien in 2006. After post‐doctoral stays in Belfast, UK and Copenhagen, DK, she returned to TU Wien to establish her independent research career focused on green and sustainable chemistry. In 2021, she was appointed Full Professor for Sustainable Chemistry. Katharina Schröder's research interests are based on sustainable organic chemistry, with a special focus on (i) novel catalytic processes for asymmetric synthesis, (ii) on carbon capture and valorization techniques (CCU), particularly on photocatalytic CO_2_ activation and (iii) on the recovery of valuable ingredients from industrial waste streams using advanced fluid technologies*.